# External damp environment aggravates diarrhea in spleen deficiency and dampness syndrome in mice: involvement of small intestinal contents microbiota, energy metabolism, gastrointestinal and fluid functions

**DOI:** 10.3389/fcimb.2024.1495311

**Published:** 2024-10-31

**Authors:** Donglin Yu, Shiqin Xie, Mingmin Guo, Yi Wu, Qianghong Tian, Zhiyan Wang, Sainan Zhou, Ying Cai

**Affiliations:** ^1^ College of Chinese Medicine, Hunan University of Chinese Medicine, Changsha, Hunan, China; ^2^ College of Pharmacy, Hunan University of Chinese Medicine, Changsha, Hunan, China; ^3^ The First Affiliated Hospital of Hunan University of Chinese Medicine, Changsha, Hunan, China

**Keywords:** external damp environment, intestinal microbiota, spleen deficiency and dampness, diarrhea, multiplatform water environment, lard gavage, renal-intestinal axis

## Abstract

**Objectives:**

Recent studies have increasingly demonstrated that a multiplatform water environment combined with lard gavage is an effective method for establishing a mouse model of diarrhea. However, the interactions between intestinal microorganisms and diarrhea, as well as the relationships among energy metabolism, fluid balance, and gastrointestinal function in this model, remain poorly understood.

**Methods:**

Building on previous research, this study aimed to optimiz and replicate a multiplatform water environment combined with a lard gavage model. Male Kunming mice, free of specific pathogens, were randomly divided into four groups: a normal control group (ZC), a standing group (ZL), a standing combined with lard group (ZLZ), and a standing combined with internal and external wet conditions group (ZLZS). The mice in the ZL, ZLZ, and ZLZS groups were subjected to 4 hours of daily standing in a custom-designed multiplatform water environment. Starting on day 8, mice in the ZLZ and ZLZS groups were gavaged with lard (0.4 mL per session, twice daily) for 7 consecutive days, while those in the ZLZS group were additionally exposed to a wet litter environment (50 g/100 mL). The ZC and ZL groups received equal volumes of sterile water via gavage. The microbiota in the small intestine, as well as serum levels of cAMP, cGMP, VIP, Gas, and D-xylose, were analyzed.

**Results:**

Compared with the ZLZ group, the ZLZS group showed significantly lower serum levels of cAMP/cGMP (*p*<0.01) and Gas (*p*<0.01). D-xylose levels were lower in the ZL, ZLZ, and ZLZS groups compared to the ZC group, while VIP levels were significantly higher in the ZL and ZLZS groups (*p*<0.01). Moverover, *Corynebacterium*, *Empedobacter*, and *Pseudochrobactrum* were identified as characteristic bacterial genera in the ZLZS group. The mechanism by which the small intestinal microbiota induces diarrhea was linked to the biosynthesis of secondary bile acids.

**Conclusion:**

A multiplatform water environment combined with lard gavage can effectively induce diarrhea, and the addition of an external wet environment exacerbates this condition by affecting small intestinal contents microbiota and other functions.

## 1 Introduction

Diarrhea is a common symptom of gastrointestinal diseases, often associated with disorders in gastrointestinal motility, intestinal microbiota imbalances, impaired intestinal barrier function, and disturbances in fluid homeostasis. These disturbances are mediated by the neuroendocrine-immune network and intestinal smooth muscle dysfunction (Bai et al., 2021). The combination of a multi-platform water environment and lard gavage is a widely used method for inducing diarrhea in mouse models. Studies suggest that the multi-platform water environment disrupts rapid eye movement (REM) sleep in mice, inducing central fatigue (Sharma et al., 2021). Additionally, a high-fat diet, such as lard, creates an inflammatory and immunosuppressive tumor microenvironment, inhibiting macrophage mitochondrial uptake and altering metabolic homeostasis under pathological conditions (Tong et al., 2021; Borcherding et al., 2022). In traditional Chinese medicine (TCM), these conditions are linked to “dampness,” while modern medicine considers humidity one of the four essential elements of human life (Qiu et al., 2020). However, the external damp environment in the multi-platform water and lard gavage model does not produce sustained effects in mice. To address this, we optimized the model by incorporating a damp bedding factor, allowing us to explore the intestinal microbiota in small intestinal contents and their functional correlations.

The intestinal microbiota plays an essential role in host metabolism, immunity, and health, particularly in the development of innate and adaptive immune systems, which maintain the symbiotic relationship between the host and its microbiota (Ma et al., 2022). Overgrowth of both non-resident and resident microbiota in the small intestine can contribute to diarrhea. Intestinal microbiota dysbiosis leads to gastrointestinal mucosal stress, triggering the release of gastrointestinal hormones by enteric neurons, which increases mucosal water and electrolyte secretion while promoting gastrointestinal motility during digestion (Li et al., 2022a; Mu, 2023). The intestinal microbiota also plays a crucial role in energy metabolism. Colonic epithelial cells primarily derive their energy from the microbiota, and the efficiency of microbial energy extraction directly impacts cellular function (Fourie et al., 2017). Disorders such as irritable bowel syndrome (IBS) and inflammatory bowel disease (IBD) are often accompanied by reduced energy, lipid, and amino acid metabolism. Furthermore, fluid balance and the intestinal microbiota are mutually regulated. On the one hand, water is a final oxidation product of intestinal microbiota metabolism; on the other hand, fluid absorption and excretion depend on intestinal mucosal barrier permeability. Dysbiosis and abnormal permeability are key mechanisms underlying diarrhea (Tang et al., 2021).

In conclusion, this study investigates the relationships between intestinal microbiota in the small intestine, gastrointestinal function indicators such as gastrin (Gas) and D-xylose, energy metabolism markers including cyclic adenosine monophosphate (cAMP) and cyclic guanosine monophosphate (cGMP), and the fluid metabolism marker vasoactive intestinal peptide (VIP). These findings provide a scientific basis for optimizing the multi-platform water environment and lard gavage model and improving TCM-based animal models. Moreover, this study offers valuable insights into promoting healthy lifestyle practices.

## 2 Materials and methods

### 2.1 Animals

Forty SPF-grade, 4-week-old male Kunming mice, each weighing 20 ± 2 g, were obtained from Hunan Slake Jingda Experimental Animal Co., Ltd. [Animal Experimental Qualification Number: SCXK (Xiang) 2019–0004] (Wu et al., 2022). The mice were housed at the Experimental Animal Center of Hunan University of Chinese Medicine [SCXK (Xiang) 2019–0009], where they were maintained at a room temperature of 23–25°C and relative humidity of 47–53%, with a light cycle from 07:00–17:00 daily and complete darkness from 17:00–07:00 the following day. The environment was clean and quiet. This experiment was reviewed and approved by the Experimental Animal Ethics Committee of Hunan University of Chinese Medicine, with ethics number HNUCM21-2404-29.

### 2.2 Feed

The breeding feed used for the mice in this study was provided by the Experimental Animal Center of Hunan University of Chinese Medicine and manufactured by Beijing Huafukang Biotechnology Co., Ltd. The main ingredients of the feed included crude protein ≥20%, crude fat ≥4%, crude fiber ≤5%, crude lime ≤8%, moisture ≤10%, lysine ≥1.3%, calcium 0.6%-1.8%, phosphorus 0.6%-1.2%, and salt 0.3%-0.8%, ensuring cleanliness and freedom from contamination. The Beijing feed certificate number for the feed is (2019) 06076. Jinluo refined lard (Liu et al., 2023), produced by Linyi Xincheng Jinluo Meat Products Group Co., Ltd. (Production License No. SC10337130200099, Product Standard No. GB10146), contains energy (44%) and fat (167%) as its main nutrients. Before use, the lard was heated in a water bath until it melted and then gavaged at 37°C.

### 2.3 Main experimental equipment

A custom-designed water environment with a small platform standing box measuring 112 cm × 62 cm × 41 cm was constructed (Wang, 2022; Qiao et al., 2024). The box contained twelve small platforms, each with a diameter of 3 cm and a height of 12 cm. The platforms were spaced more than 10 cm apart. Water was filled around the platforms, with the water surface approximately 1 cm below the platform surface. The water temperature was maintained at 25 ± 2°C.

### 2.4 Grouping and modeling methods

**Figure 1:**
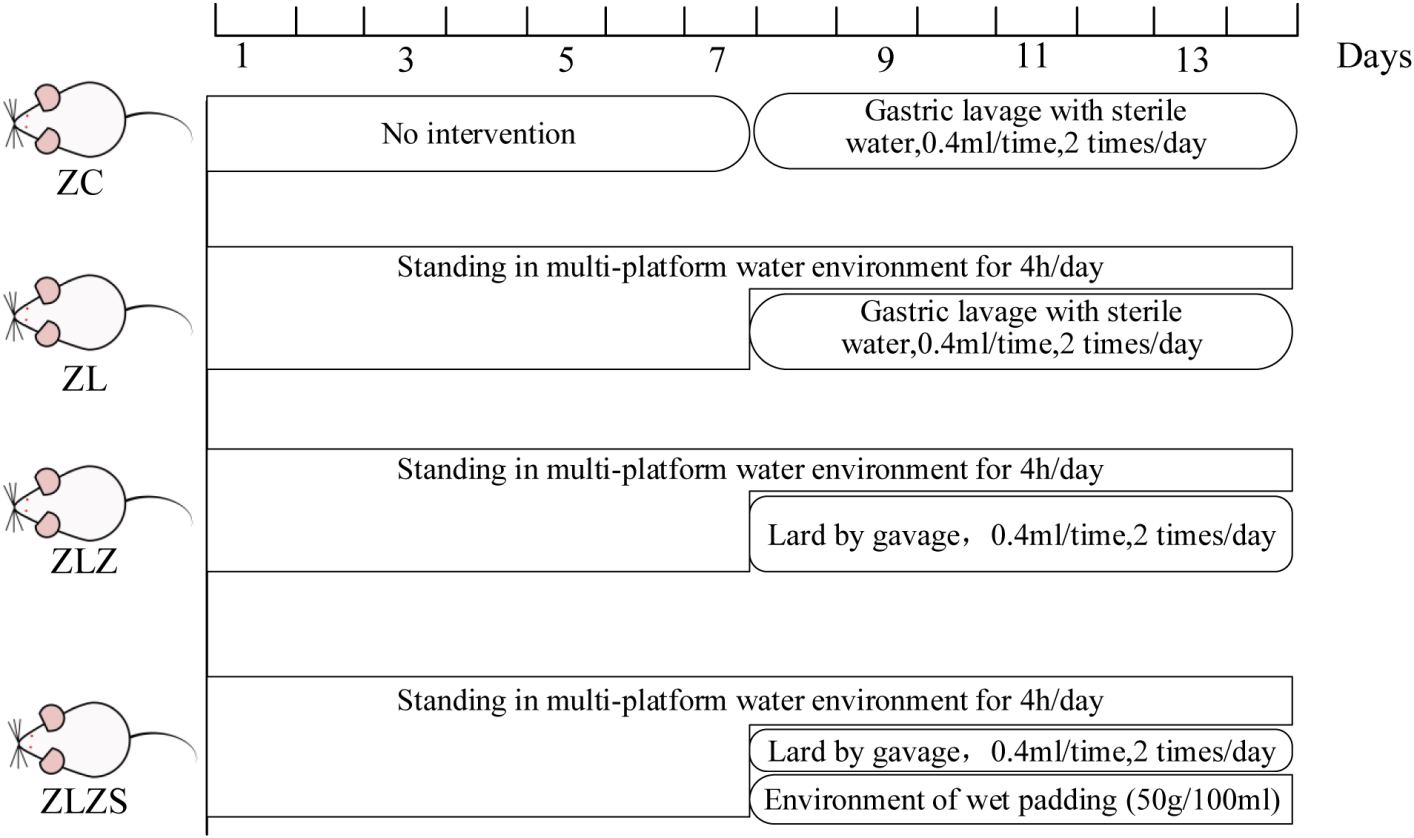
Experimental flow chart.

After 3 days of acclimatization, 40 mice were randomly assigned to four groups: a normal group (ZC), a standing group (ZL), a standing combined with lard group (ZLZ), and a standing combined with internal and external wet group (ZLZS), with 10 mice in each group. The experimental procedures followed the methods described in the literature [see (Liu et al., 2023)]. The mice in the ZL group were placed on a small platform within a custom-designed water environment for 4 hours daily for the first 7 days and then gavaged with 0.4 mL of distilled water twice daily from the 8th day onward. Mice in the ZLZ group were subjected to the same standing protocol as those in the ZL group but were gavaged with 0.4 mL of lard twice daily starting on the 8th day. Mice in the ZLZS group followed the ZL protocol, but from the 8th day onward, they were gavaged with 0.4 mL of lard twice daily and exposed to a wet bedding environment (50 g/100 mL) (Zhu et al., 2021). The mice in the ZC group were not subjected to the standing protocol and had ad libitum access to food and water. On the 8th day, they were gavaged with the same volume of standard sterile water. Each group continued with their respective treatment for a total of 14 days. The specific experimental flow chart is shown in **Figure 1**.

### 2.5 Evaluation of the diarrhea model of spleen deficiency and dampness excess syndrome

#### 2.5.1 General conditions

The general characteristics of the mice were observed at 8:30 AM on the 1st, 8th, and 14th days of the experiment. The observations included the mental state, activity level, coat condition, and fecal output of the mice. Additionally, the body weights of the mice in each group were recorded on the 1st, 4th, 7th, 11th, and 14th days.

#### 2.5.2 Determination of food intake and water intake

The food and water intake of the mice were recorded on the 1st, 8th, and 14th days of the experiment. Weighed feed was provided at 9:00 AM each day, and the remaining feed in each cage was weighed after 24 hours (Qiao et al., 2023). The average daily food intake was calculated via the following formula:


$$Average\;daily\;food\;intake=\frac{Amount\;of\;feed\;placed-Amount\;of\;feed\;remaining\;after\;24\;hours}{Number\;of\;mice}$$

Water intake was measured similarly. A consistent volume of drinking water was provided each morning, and the remaining volume was measured with a graduated cylinder after 24 hours. The average daily water intake was determined via the following formula:


$$Average\;daily\;water\;intake=\frac{Amount\;of\;drinking\;water\;placed-Amount\;of\;water\;remaining\;after\;24\;hours}{Number\;of\;mice}$$

#### 2.5.3 Determination of fecal moisture content and defecation frequency

The number of defecations by each mouse was recorded at 8:30 AM on the 1st, 4th, 7th, 11th, and 14th days, and the fecal moisture content was calculated. Each mouse was placed in a clean, dry cage and allowed to move freely for 30 minutes. The number of fecal particles in each cage was then counted. A single particle was counted as one defecation, and two consecutive particles were counted as one defecation. The number of defecations per mouse during this period was calculated. Feces from each mouse were collected as a sample, with 3 to 4 fecal pellets collected on weighing paper, and the wet weight of each sample was recorded (Shao et al., 2022). The collected feces were then heated and dried at 110°C until a constant weight was achieved and weighed again, and the fecal moisture content was calculated via the following formula:


$$Fecal\;moisture\;content\;\left(\%\right)=\frac{Wet\;weight-Dry\;weight}{Wet\;weight}\;\times 100\%$$

During feces collection, care was taken to absorb any urine to minimize errors.

#### 2.5.4 Determination of the serum D-xylose, Gas, VIP, cAMP and cGMP contents

Before blood collection, each mouse was weighed. Blood was collected by removing the eyeball, and the samples were allowed to stand until stratification occurred. The blood samples were then centrifuged at 4°C and 3000 r/min for 10 minutes (Zhou et al., 2024). The upper serum layer was carefully pipetted and analyzed for three key indicators of diarrhea associated with spleen deficiency and dampness: the energy index, gastrointestinal function index, and fluid metabolism index. These indicators were measured in strict accordance with the instructions provided with the ELISA kits. The concentrations of the standards were plotted on the horizontal axis, and the corresponding optical density (OD) values were plotted on the vertical axis to generate a linear regression curve. The concentration values of each sample were calculated via a curve equation. The specifications for the ELISA kits used in the analysis are as follows: Mouse Vasoactive Intestinal Peptide (VIP) ELISA Kit (Batch number: JM-02729M2, Jiangsu Jingmei Biotechnology Co., Ltd.), Mouse Gastrin ELISA Kit (Batch number: JM-03140M2, Jiangsu Jingmei Biotechnology Co., Ltd.), Mouse D-xylose ELISA Kit (Batch number: JM-12962M2, Jiangsu Jingmei Biotechnology Co., Ltd.), Mouse Cyclic Guanosine Monophosphate (cGMP) ELISA Kit (Batch number: JM-02304M2, Jiangsu Jingmei Biotechnology Co., Ltd.), and Mouse Cyclic Adenosine Monophosphate (cAMP) ELISA Kit (Batch number: JM-02827M2, Jiangsu Jingmei Biotechnology Co., Ltd.).

#### 2.5.5 Organ indices

After the mice were euthanized via cervical dislocation, they were immediately placed on a clean bench. The spleen and thymus were carefully excised, ensuring that they remained intact. Fascia and adipose tissue were removed, and blood stains on the organ surfaces were blotted using filter paper (Xie et al., 2021). The organs were then weighed, and the spleen and thymus indices were calculated as follows: Organ index(%) = Organ mass (g)/Body mass (g)×100%.

#### 2.5.6 Extraction of small intestinal contents

Small intestinal segments from five randomly selected mice in each group were isolated. The contents of the small intestine were carefully scraped using curved forceps on an ice-cold culture dish. These samples were then collected into 1.5 mLEP tubes and labeled accordingly (Wu et al., 2021). The samples were immediately sent for analysis, which was conducted by Shanghai Paisono Biotechnology Co., Ltd.

#### 2.5.7 Detection of the structure and diversity of the small intestinal microbiota via 16S rRNA

The quantity and quality of the DNA were assessed via a NanoDrop NC2000 spectrophotometer and an agarose gel electrophoresis instrument. The forward primer F: 5´-ACTCCTACGGGAGGCAGCA-3´ and the reverse primer R: 5´-GGACTACHVGGGTWTCTAAT-3´ were designed to amplify the V3+V4 region of the 16S rRNA gene via PCR. The amplified products were detected via 1.20% agarose gel electrophoresis, and the target fragment was recovered via the AxyPrep PCR Cleanup Kit. The purified PCR product was quantified via fluorescence with a Bio Tek fluorescence quantitative instrument and a Quant-iT PicoGreen dsDNA Assay Kit (Shen, 2021). The prepared DNA library was then sequenced on the PacBio Sequel platform. Dilution curves and material accumulation curves were used to evaluate the quality of the raw data. The primer fragments were removed via the *qiime cutadapt trim-pair* command, followed by quality control, denoising, splicing, and chimera removal [see (Liu et al., 2023)]. DADA2 sequence denoising, implemented in QIIME2 software, provided 100% similarity clustering, merged ASV feature sequences, and generated ASV tables while removing singleton ASVs. The classify-sklearn algorithm in QIIME2 was employed to annotate ASV feature sequences or representative OTU sequences. The resulting species information was analyzed via ASV/OTU Venn diagrams, alpha diversity, beta diversity, dominant bacterial species composition, characteristic bacterial species, and functional prediction.

The datasets used and analyzed during the current study are available from the corresponding author on reasonable request. The data presented in the study are deposited in the NCBI repository, accession number PRJNA1159995.

#### 2.5.8 Hematoxylin−eosin staining

Five small intestinal segments from each group were randomly selected, and each segment was fixed in 4% paraformaldehyde solution for 24h for routine HE staining. The fixed small intestinal tissue was dehydrated, embedded, and cut into 4 μm thick slices. The samples were then placed in xylene I, xylene II, anhydrous ethanol I, anhydrous ethanol II, and 75% alcohol for dewaxing and hydration, stained with hematoxylin solution for 3–5 min, differentiated, blued, rinsed with running water, dehydrated with gradient alcohol, and then stained with eosin solution for 5 min. The slides were sealed with neutral gum, and the collected images were observed and analyzed under a microscope (Li et al., 2024). ImageJ software was used to measure the height of the intestinal villi and the depth of the crypts in each group of mice to assess the activity and function of the intestinal stem cells (Gao et al., 2023).

#### 2.5.9 Periodic acid-Schiff staining

The remaining small intestine segments from the 5 randomly selected mice in operation 1.5.7 were fixed, paraffin-embedded, and sectioned. After dewaxing with xylene and gradient ethanol, the sections were hydrated in distilled water, treated with an oxidant for 5–10 minutes, and then rinsed with running water for 5 minutes. Schiff stain solution was applied, followed by staining, rinsing, and counterstaining with hematoxylin for 10 minutes. The slides were rinsed again, dehydrated with gradient ethanol, cleared with xylene, and sealed with neutral resin. The slides were observed under a microscope to evaluate the staining conditions (Yan, 2023). Image-Pro Plus 6.0 software was used to randomly select 8 fields of view from the intestinal tissue of each group of mice, and the number of goblet cells in the selected areas was counted (Yang et al., 2021). The number of goblet cells per unit area in each group was calculated to assess the fluid metabolism capacity of the intestinal tissue.

#### 2.5.10 Determination of the intestinal water content

After blood collection, the mice were euthanized by cervical dislocation. Following dissection, approximately 3 cm of the colon was excised. The intestine was opened, and food residues and residual blood were washed away with saline. The intestine was then blotted dry with filter paper and placed on a clean sheet of filter paper. The weight of the intestine was measured and recorded. The intestine was subsequently placed in an oven at 110°C for 8 hours (Wang et al., 2024). After drying, the intestine, along with the filter paper, was weighed again. The water content of the intestine was calculated via the following formula:


$$Water\;content=\frac{Wet\;mass-Dry\;mass}{Wet\;mass}\times 100\%$$

### 2.6 Statistical methods

The data were analyzed via SPSS 25.0 software. The measured data for each group were assessed for a normal distribution and are expressed as the mean ± standard deviation ( $\bar{x}\;\pm \;SD$ ). For data with equal variances, one-way ANOVA was employed for comparisons across groups, followed by the least significant difference (LSD) method for pairwise comparisons. In cases where variances were unequal, Welch’s approximate F test was used for multiple group comparisons, and Dunnett’s T3 method was applied for pairwise comparisons. Data not conforming to a normal distribution are expressed as medians with interquartile ranges and were analyzed via the nonparametric Kruskal−Wallis *H* test. The significance level was set at α=0.05.

## 3 Results

### 3.1 Comparison of the macroscopic signs of the mice in each group

**Figure 2:**
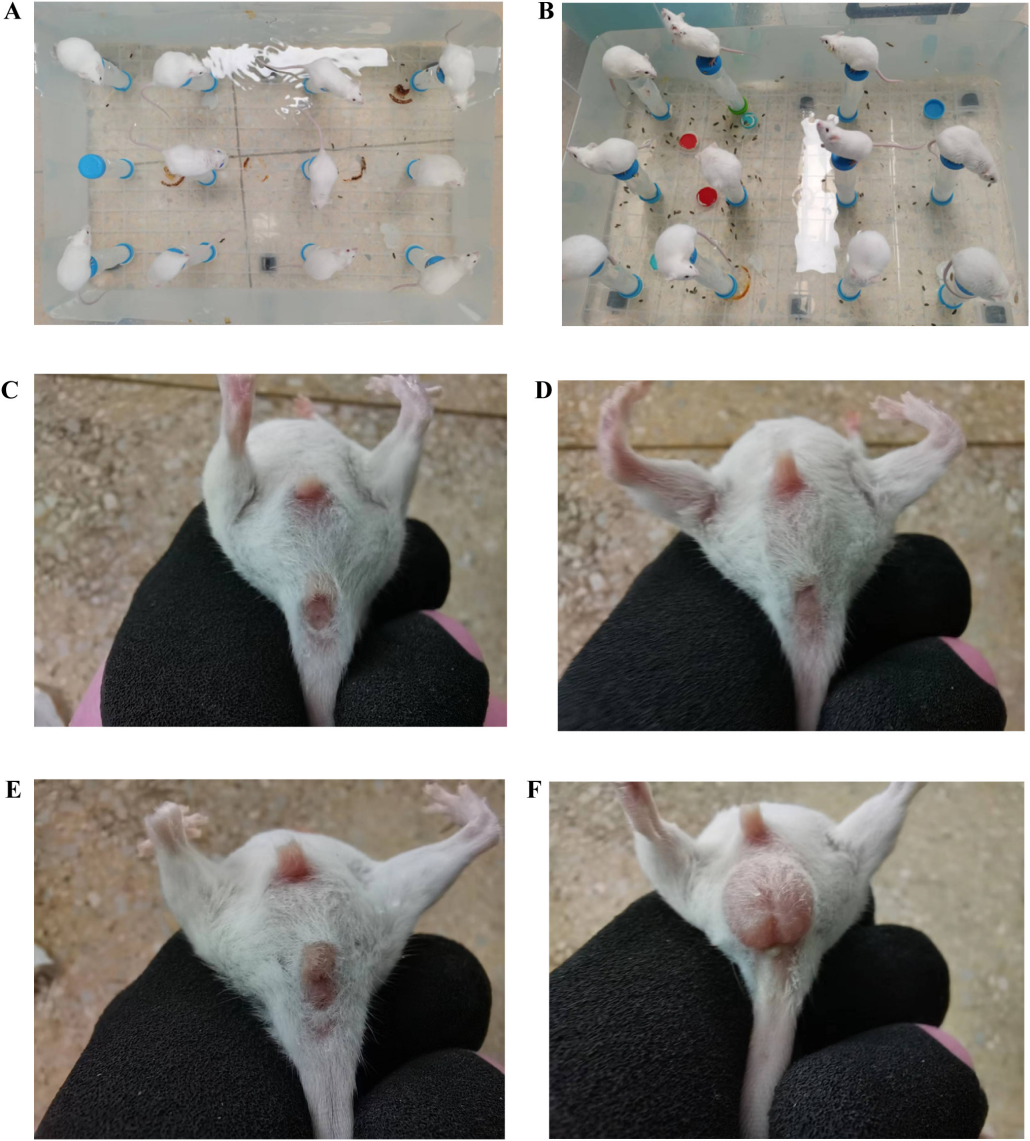
General characteristics of the mice in each group on the 11th day of the spleen deficiency and dampness diarrhea model. **(A, B)** Homemade water environment small platform device. **(C)** Analyses and perianal conditions of the mice in the ZC group. **(D)** Analyses and perianal conditions of the mice in the ZL group. **(E)** Analyses and perianal conditions of the mice in the ZLZ group. **(F)** Analysis and perianal conditions of the mice in the ZLZS group.

**Figure 3:**
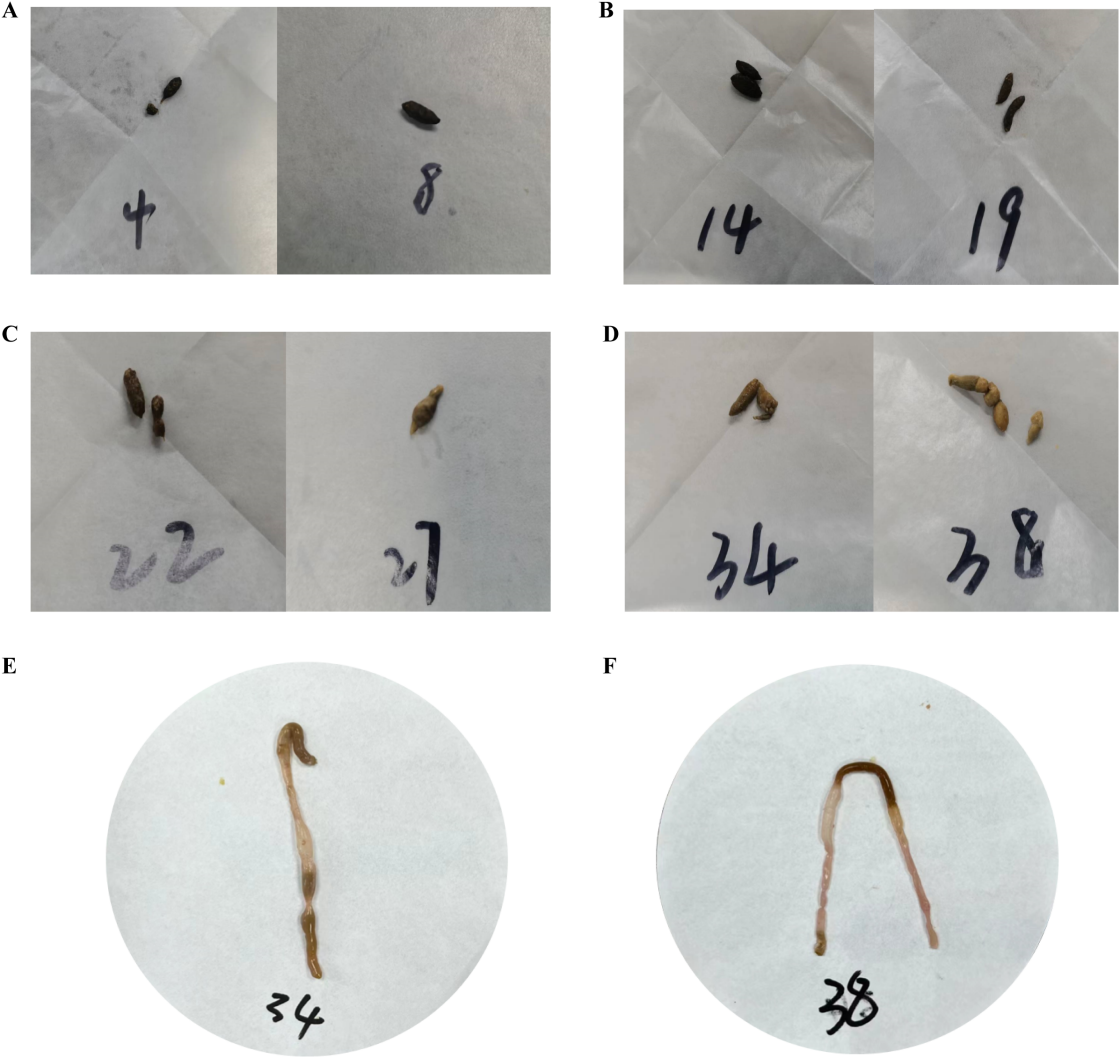
Fecal characteristics of the mice in each group on the 11th day of modeling. **(A)** Feces of mice in the ZC group. **(B)** Feces of mice in the ZL group. **(C)** Feces of mice in the ZLZ group. **(D)** Feces of mice in the ZLZS group. **(E)** Mouse No. 34 in the ZLZS group exhibited gastrointestinal bloating. **(F)** Mouse No. 38 in the ZLZS group exhibited gastrointestinal bloating.

After modeling, the mice in the ZC group exhibited a quick response to stimuli, with smooth fur and black, dry feces shaped like rice grains, which were firm and did not stick to paper when lightly pressed. In the ZL group, food intake was greater than that in the ZC group, but body weight was lower. The fur of the mice appeared messy, and although their mental state was stable, their feces were black, relatively moist, and deformed when pressed. The ZLZ group showed slower reactions and messy fur. Initially, their food and water intakes were similar to those in the ZL group, but both significantly decreased in the later stages. The weight gain of the mice in this group was noticeable, with lighter-colored feces that were moist and sticky. In the ZLZS group, the mice exhibited slow reactions and deteriorating mental states, with messy fur. Food and water intake were not significantly different from those in the ZLZ group, but their feces were lighter in color, moist, and accompanied by continuous defecation (as shown in **Figures 2**, **3A–D**).

As shown in **Figures 3E** and **F**, the mice in the ZLZS group exhibited varying degrees of gastrointestinal bloating. These findings may be attributed to the excessive dampness characteristic of the spleen deficiency and dampness excess model, which potentially obstructs the Qi mechanism.

**Figure 4:**
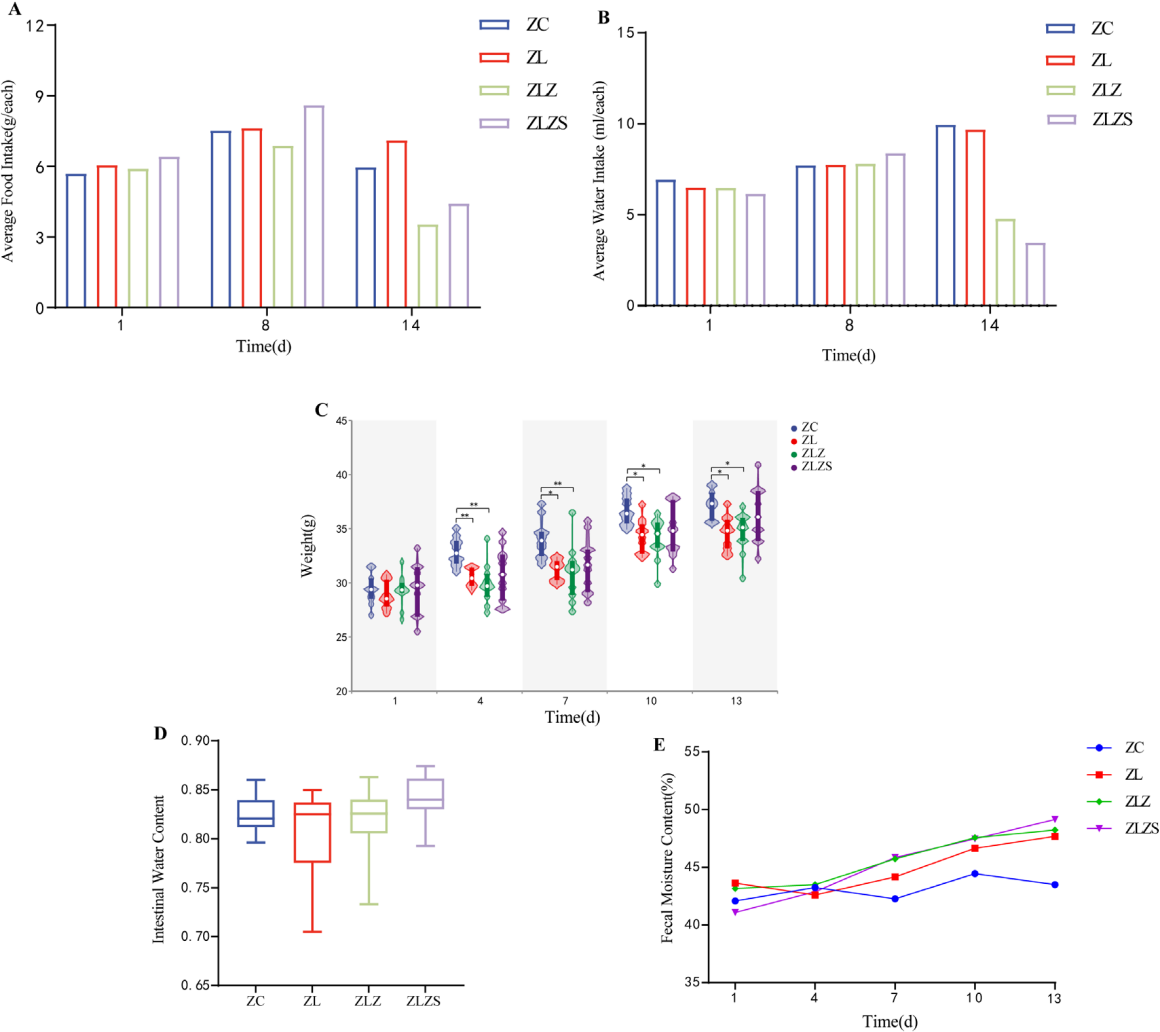
General characteristics of mice with diarrhea due to spleen deficiency and dampness. **(A)** Average food intake. **(B)** Average water intake. **(C)** Body weight. **(D)** Intestinal water content. **(E)** Fecal water content. (^*^ *p*<0.05, ^**^ *p*<0.01, ZC normal group, ZL standing group, ZLZ standing combined with lard group, ZLZS standing combined with internal and external dampness group).

On the first day of modeling, the average food and water intakes of the groups were similar (**Figures 4A, B**). By the eighth day, both food and water intake had increased across all the groups, with the ZL, ZLZ, and ZLZS groups consuming more water than the ZC group did. After seven days of lard gavage, the food intake of the ZC group remained stable, whereas that of the ZL group continued to increase, albeit less than that on the eighth day. However, the ZLZ and ZLZS groups experienced a significant decrease in food intake. By the fourteenth day, the ZC and ZL groups presented increased water intake, whereas the ZLZ and ZLZS groups presented a decrease, most notably in the ZLZS group.

Seven days before modeling, the weight difference between the ZL group and the ZC group gradually decreased over time (*p*<0.05). However, the weight difference between the ZLZ group and the ZC group was more pronounced (*p*<0.01). After seven days of modeling, the weight gain in the ZLZS group exceeded that in the ZC group, with the significant difference between the ZC and ZL groups remaining stable (*p*<0.05), whereas the difference from the ZLZ group decreased (p<0.05). Notably, before modeling, the body weight trends in the ZL, ZLZ, and ZLZS groups were consistent. After the introduction of an external damp environment, the body weights of the ZL and ZLZ groups decreased below those of the ZC group, whereas those of the ZLZS group continued to increase (**Figure 4C**). These findings suggest that while standing combined with lard gavage induced weight loss in the model mice, the addition of an external damp environment inhibited this effect. These findings align with the clinical diagnosis of spleen deficiency and dampness syndrome, where environmental factors play a significant role in symptom manifestation.

As shown in **Figure 4E**, the fecal water content of the mice in the ZLZS group gradually increased with increasing duration of the modeling period. Starting on the eighth day, the ZLZ and ZLZS groups presented higher fecal water contents than did the other groups. Additionally, as depicted in **Figure 4D**, the intestinal water content in the ZLZS group was greater than that in the other modeling groups. These results indicate that the combination of standing and lard gavage can induce diarrhea in mice and that the presence of external moisture factors exacerbates pathological symptoms.

### 3.2 Effects of diarrhea caused by spleen deficiency and dampness syndrome on immunity and energy metabolism in mice

**Figure 5:**
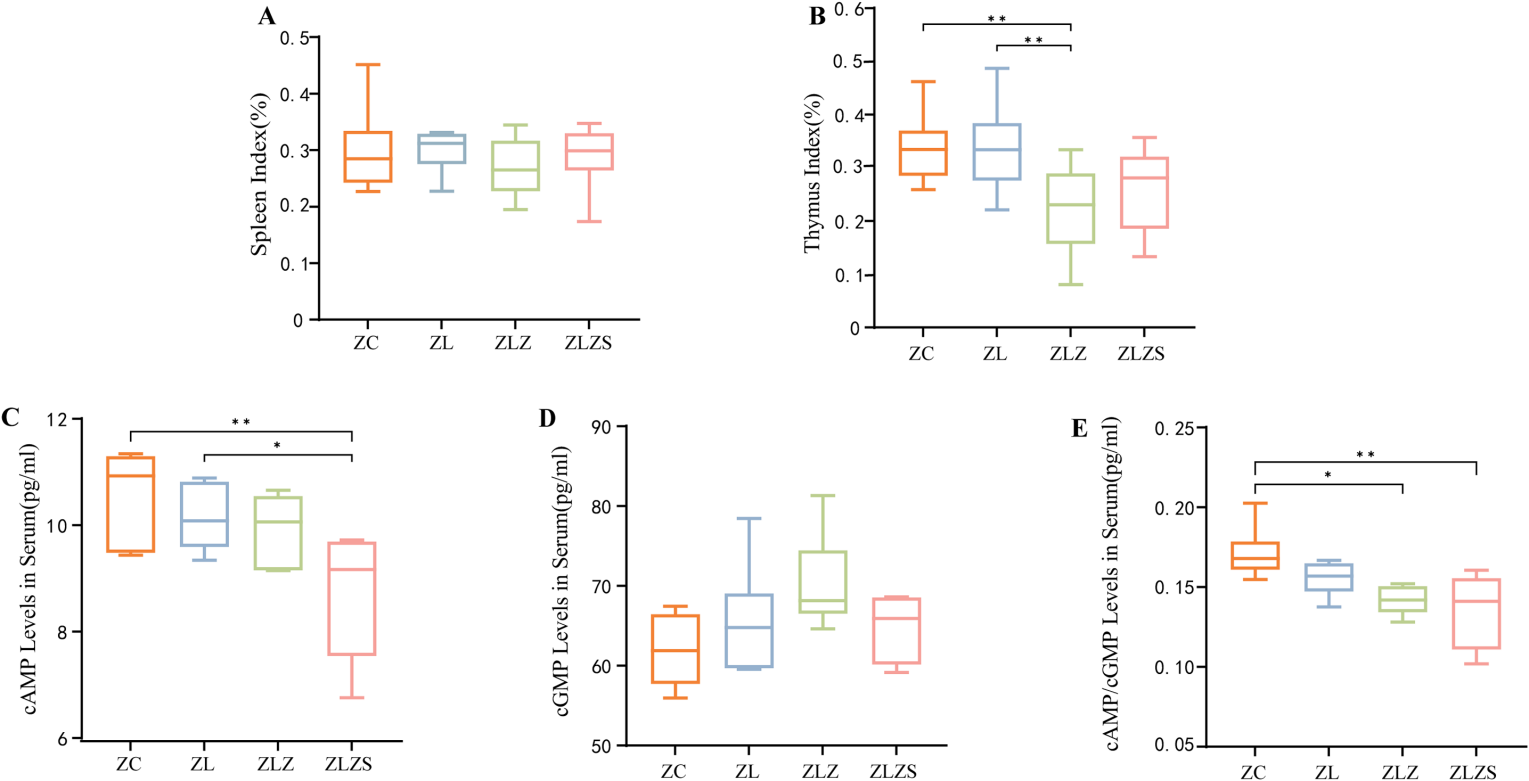
Box plots of organ indices for mice across different groups, where the organ index is calculated as the ratio of organ weight to mouse weight. Panel **(A)** Shows the spleen index, and panel **(B)** Displays the thymus index. Panel **(C)** shows the serum levels of cyclic adenosine monophosphate (cAMP), while panel **(D)** Shows the serum levels of cyclic guanosine monophosphate (cGMP). Panel **(E)** Shows the cAMP/cGMP ratio. Significant differences are indicated with ^*^ *p*<0.05 and ^**^ *p*<0.01. The groups are as follows: ZC (normal group), ZL (standing group), ZLZ (standing combined with lard group), and ZLZS (standing combined with internal and external moisture groups).

The spleen and thymus are critical immune organs whose indices provide indirect measures of the host’s immune function. As shown in **Figures 5A** and **B**, postmodeling analysis revealed no significant changes in the spleen index across the groups (*p*>0.05). However, the thymus index in the ZLZ group was significantly lower than that in the ZC and ZL groups (*p*<0.01). These findings indicate that the combination of the multiplatform water environment and lard gavage notably impaired immune function in mice, with a more pronounced effect on the thymus than on the spleen.

As shown in **Figure 5C**, the serum cyclic adenosine monophosphate (cAMP) levels in the ZLZS group were significantly lower than those in the ZC group (*p*<0.01). Conversely, the serum cAMP levels in the ZL group were significantly greater than those in the ZLZS group (*p*<0.05). **Figure 5D** shows an increasing trend in serum cyclic guanosine monophosphate (cGMP) across all groups, although this trend was not statistically significant. Furthermore, as depicted in **Figure 5E**, the cAMP/cGMP ratio in the model groups was significantly lower than that in the ZC and ZLZ groups (*p*<0.05 for ZLZ and *p*<0.01 for ZLZS).

### 3.3 Effects of diarrhea caused by spleen deficiency and dampness syndrome on gastrointestinal function and fluid metabolism in mice

**Figure 6:**
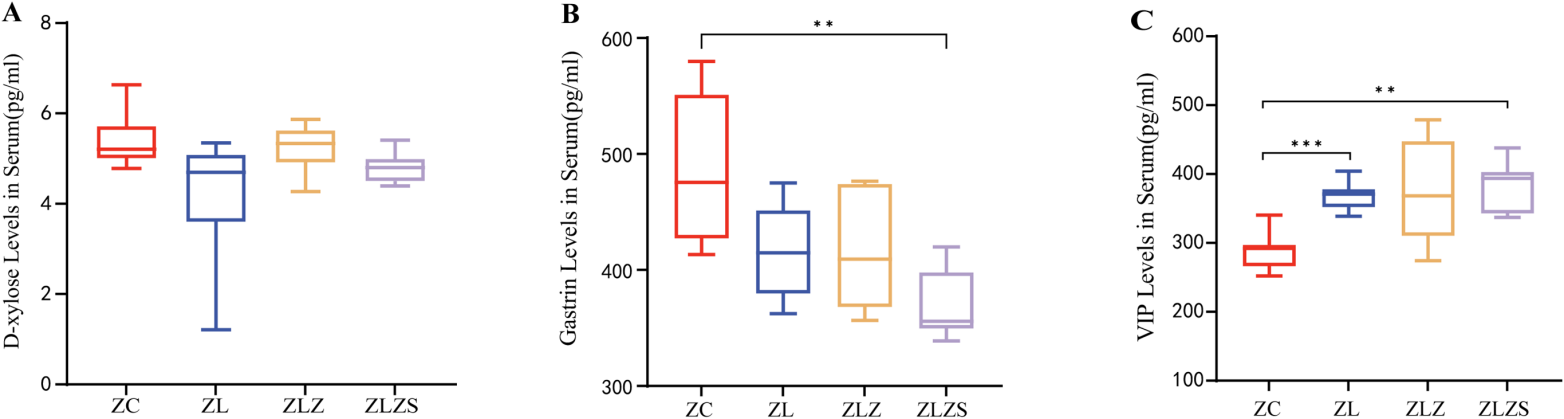
Differences in gastrointestinal function and fluid metabolism in diarrhea patients with spleen deficiency and dampness. **(A)** Serum D-xylose levels. **(B)** Serum gastrin (Gas) levels. **(C)** Serum vasoactive intestinal peptide (VIP) levels. Statistical significance is indicated as follows: **p<0.01,***p<0.001. The experimental groups included ZC (normal group), ZL (standing group), ZLZ (standing combined with lard group), and ZLZS (standing combined with internal and external dampness group).

Diarrhea resulting from fatigue combined with a high-fat diet leads to disturbances in fluid balance and reduced digestive system function. Consequently, assessing the water environment and gastrointestinal function is crucial for evaluating pathological changes. Serum D-xylose and gastrin (Gas) serve as the “gold standard” indicators for assessing gastrointestinal function in patients with spleen deficiency and dampness syndrome. As shown in **Figure 6A**, the serum D-xylose levels in the ZL, ZLZ, and ZLZS groups tended to decrease, although the differences were not statistically significant. As shown in **Figure 6B**, the Gas levels were lower in all the experimental groups than in the control group, with a statistically significant reduction in the ZLZS group compared with the ZC group (*p*<0.01). Vasoactive intestinal peptide (VIP) is a key hormone involved in fluid metabolism that influences water and salt secretion and intestinal mucosal stimulation. As shown in **Figure 6C**, postmodeling, the serum VIP levels increased across all experimental groups. Compared with those in the ZC group, the VIP levels in the ZL group were significantly different (*p*<0.001), and the ZLZS group also presented a significant difference in VIP content (*p*<0.01). These findings indicate that an external damp environment exacerbates gastrointestinal dysfunction and disrupts fluid metabolism.

### 3.4 Effects of diarrhea caused by spleen deficiency and dampness on the structure and function of the small intestine in mice

**Figure 7:**
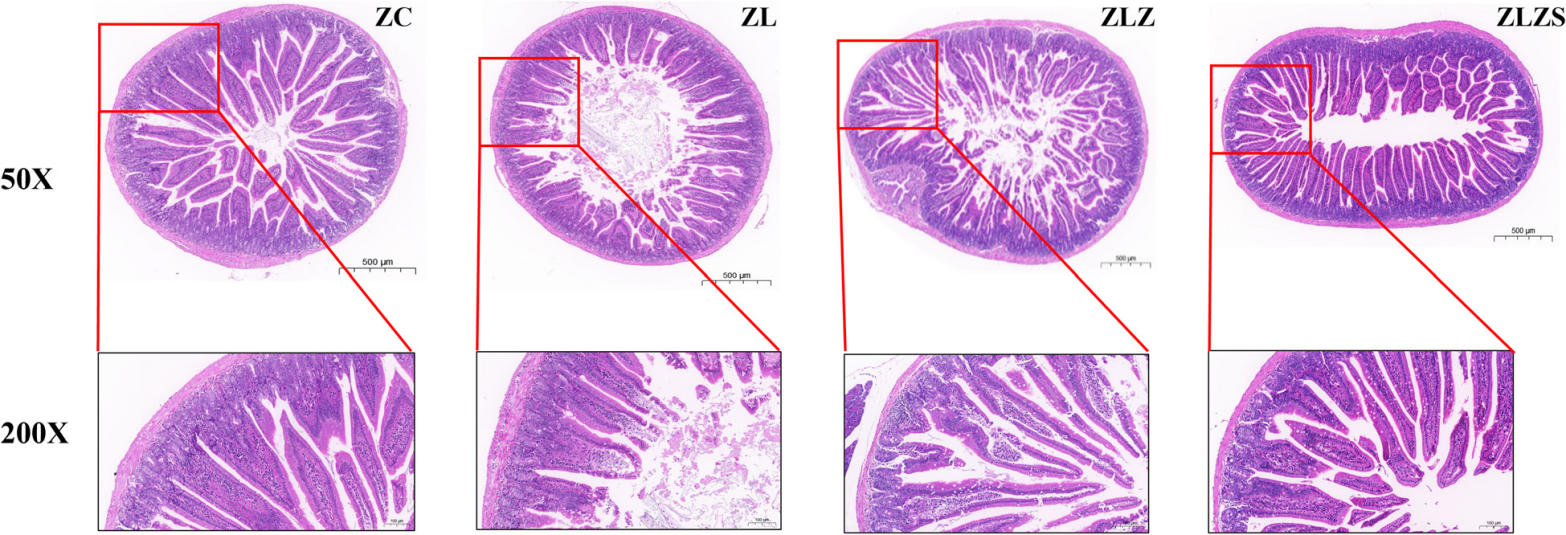
Changes in the structure and function of the small intestine.

**Figure 7** illustrates the structural changes in the small intestine of mice across different experimental groups. In the ZC group, the small intestine structure was normal, with intact mucosal lamina propria and well-preserved small intestinal villi and crypts. In contrast, the ZL group presented atrophied and flattened small intestinal villi. The ZLZ group presented more pronounced alterations, including sparse villi, a reduced number of columnar and goblet cells on the villi surface, and a thinner mucosal epithelial cell layer. Compared with the ZC group, the ZLZS group presented severe structural defects, with significantly damaged and degenerated small intestinal villi.

**Table 1** presents the histopathological observations from HE staining. There were no significant differences in the morphological structure of the small intestine between the ZLZ and ZLZS groups (*p*>0.05). However, differences were noted in the villus height, crypt depth, and the villus height/crypt depth ratio among the groups. Specifically, the height of the small intestinal villi in both the ZLZ and ZLZS groups was significantly lower than that in the ZC group (*p*<0.01). Additionally, the crypt depth in the ZLZS group was significantly greater than that in both the ZC group (*p*<0.001) and the ZL group (*p*<0.01). The villus height/crypt depth ratio also differed significantly among the groups: the ZL group presented a significant difference compared with the ZC group (*p*<0.01), whereas the ZLZ group presented an extremely significant difference from the ZLZS group (*p*<0.001). According to the PAS histopathological observations, the number of goblet cells per unit area was significantly lower in the ZL group than in the ZC group (*p*<0.01), and the ZLZS group also had a lower number of goblet cells than the ZC group did, although the difference was not statistically significant (*p*>0.05).

**Table 1:** Histopathological microscopic parameters of the small intestine in mice from Each group by HE and PAS staining ( $\bar{x}$ ±SD).

Group	N	HE histopathological observation			Histopathological observation of PAS
		Fluff height(μm)	Crypt depth(μm)	Villus height/Crypt depth	Goblet cells per unit area(Pieces/μm^2^)
ZC	5	447.691 ± 78.394	109.953 ± 19.159	4.196 ± 1.145	1.820×10^-4^ ± 9.032×10^-5^
ZL	5	379.996 ± 62.141^*^	116.745 ± 17.898	3.295 ± 0.552^**^	1.233×10^-4^ ± 6.179×10^-5 **^
ZLZ	5	374.844 ± 163.040^**^	125.942 ± 24.905^**^	3.077 ± 1.385^***^	1.485×10^-4^ ± 8.598×10^-5^
ZLZS	5	376.100 ± 80.387^**^	131.542 ± 23.851^***##^	2.920 ± 0.689^***^	1.362×10^-4^ ± 9.366×10^-5 *^
*F*		3.543	5.691	9.631	3.615
*P*		0.017	0.001	0.000	0.015

Compared with the normal group, ^*^ *p* < 0.05 indicates significant differences, ^**^ *p* < 0.01 indicates highly significant differences, and ^***^ *p* < 0.001 indicates extremely significant differences. Compared with the standing group, ^##^ *p* < 0.01 indicates highly significant differences.

### 3.5 Effects of diarrhea caused by spleen deficiency and dampness syndrome on the quantity and diversity of microbiota in the small intestine of mice

**Figure 8:**
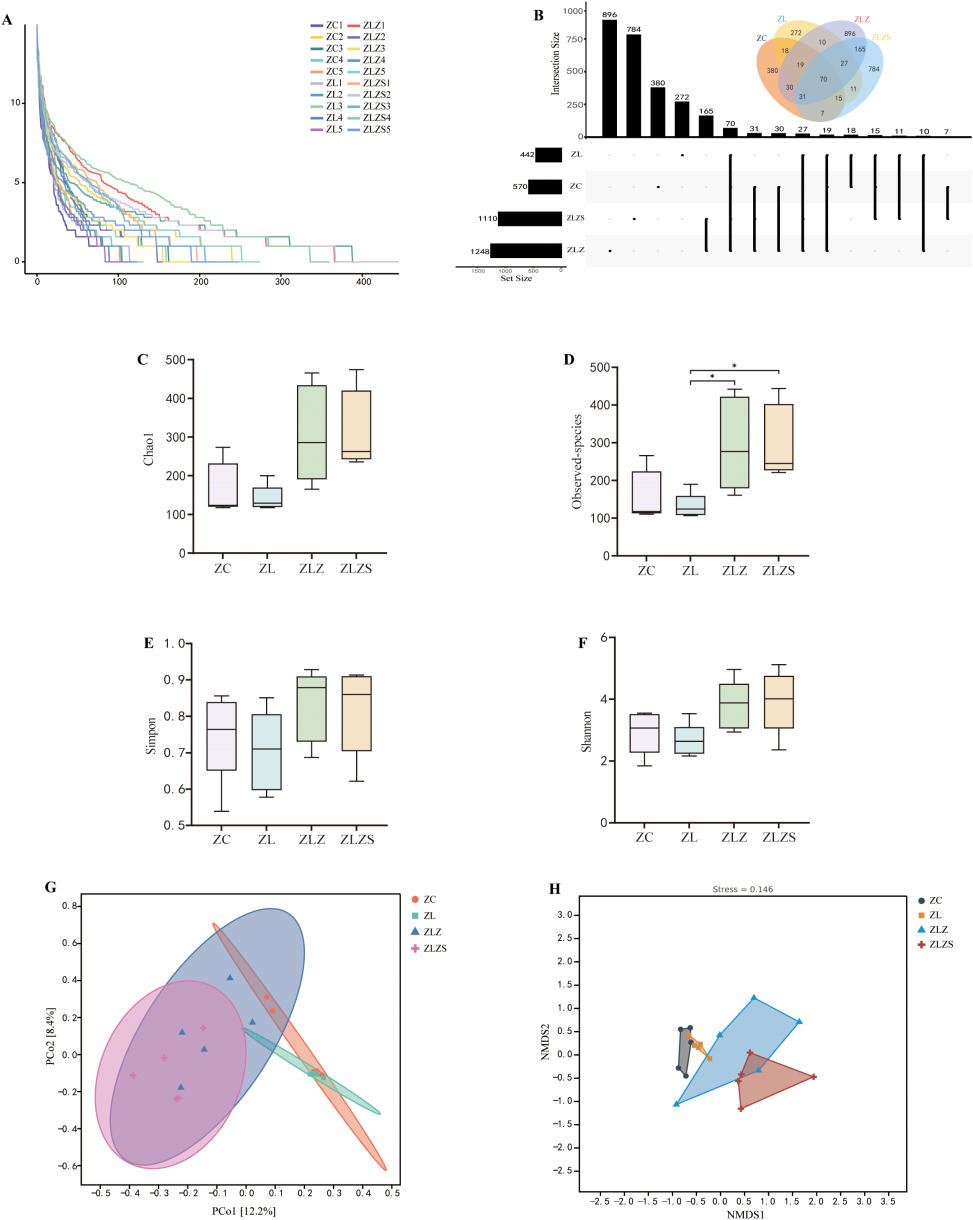
Analysis of the microbiota structure in the small intestine contents. Panel **(A)** Rarefaction curves showing the accumulation of observed ASVs/OTUs across different sample sizes. Panel **(B)** Shows the ASV/OTU Venn diagram, highlighting the unique and shared ASVs among the experimental groups. Panels **(C)** through **(F)** display the diversity indices, including the Chao1 index **(C)**, Observed-species index **(D)**, Simpson index **(E)**, and Shannon index **(F)**, which assess the richness, evenness, and diversity of the microbiota. Panel **(G)** The distance matrix and principal coordinate analysis (PCoA). Panel **(H)** Nonmetric multidimensional scaling (NMDS) analysis. Statistical significance is indicated as follows: *p<0.05

As illustrated in **Figure 8A**, the alpha diversity analysis, represented by the abundance grade curve, revealed that the horizontal axis lengths for the ZLZ and ZLZS groups were greater than those for the other groups, indicating a greater number of abundant ASVs/OTUs in these groups. Compared with those of the ZC and ZL groups, the curves for the ZLZ and ZLZS groups are relatively flatter, suggesting a smaller variance in ASV/OTU abundance and greater compositional uniformity. **Figure 8B** presents the ASV/OTU Venn diagram, which reveals that the ZC group harbors 570 ASVs, with 380 unique ASVs; the ZL group has 442 ASVs, including 272 unique ASVs; the ZLZ group possesses 1,248 ASVs, of which 896 are unique; and the ZLZS group contains 1,110 ASVs, with 784 unique ASVs. Alpha diversity, which reflects intrahabitat diversity, reflects differences in species richness, diversity, and evenness within local habitats. As shown in **Figures 8C–F**, the Chao1, observed species, Shannon, and Simpson indices for the ZLZ and ZLZS groups were greater than those for the ZC group, although these increases were not statistically significant (*p*>0.05). Conversely, the indices for the ZL group were slightly lower but not significantly different (*p*>0.05). The differences in the Chao1, Observed species, Shannon, and Simpson indices between the ZLZ and ZLZS groups were not statistically significant (*p*>0.05), yet the observed species coefficients in these groups were significantly different from those in the ZL group (*p*<0.05). Further analysis of beta diversity was conducted via principal coordinate analysis (PCA), as depicted in **Figure 8G**, where PCo1 accounted for 12.2% and PCo2 accounted for 8.4% of the variance. The samples from the ZC and ZL groups clustered closely, predominantly in the first and fourth quadrants, whereas the ZLZ and ZLZS groups presented greater similarity, primarily occupying the second and third quadrants. The closer proximity of samples in PCA indicates greater compositional similarity. **Figure 8H** shows the results of nonmetric multidimensional scaling (NMDS) analysis, with a stress value of 0.146 (less than 0.2), confirming the reliability of the results. The NMDS plot indicates that the ZC group significantly differed from the ZLZ and ZLZS groups. The spatial distribution of the ZL group is relatively concentrated, whereas the ZLZ group has the broadest distribution, with noticeable distances between samples from the ZLZ and ZLZS groups.

The structure of the intestinal microbiota significantly changed when a multiplatform water environment combined with lard gavage was used to establish a diarrhea model for spleen deficiency and dampness syndrome. Additionally, in terms of bacterial microbiota abundance, there was a notable difference between the combined internal and external wet environments and the simple lard gavage modeling methods.

### 3.6 Effects of diarrhea caused by spleen deficiency and dampness on the composition of the small intestinal microbiota in mice

**Figure 9:**
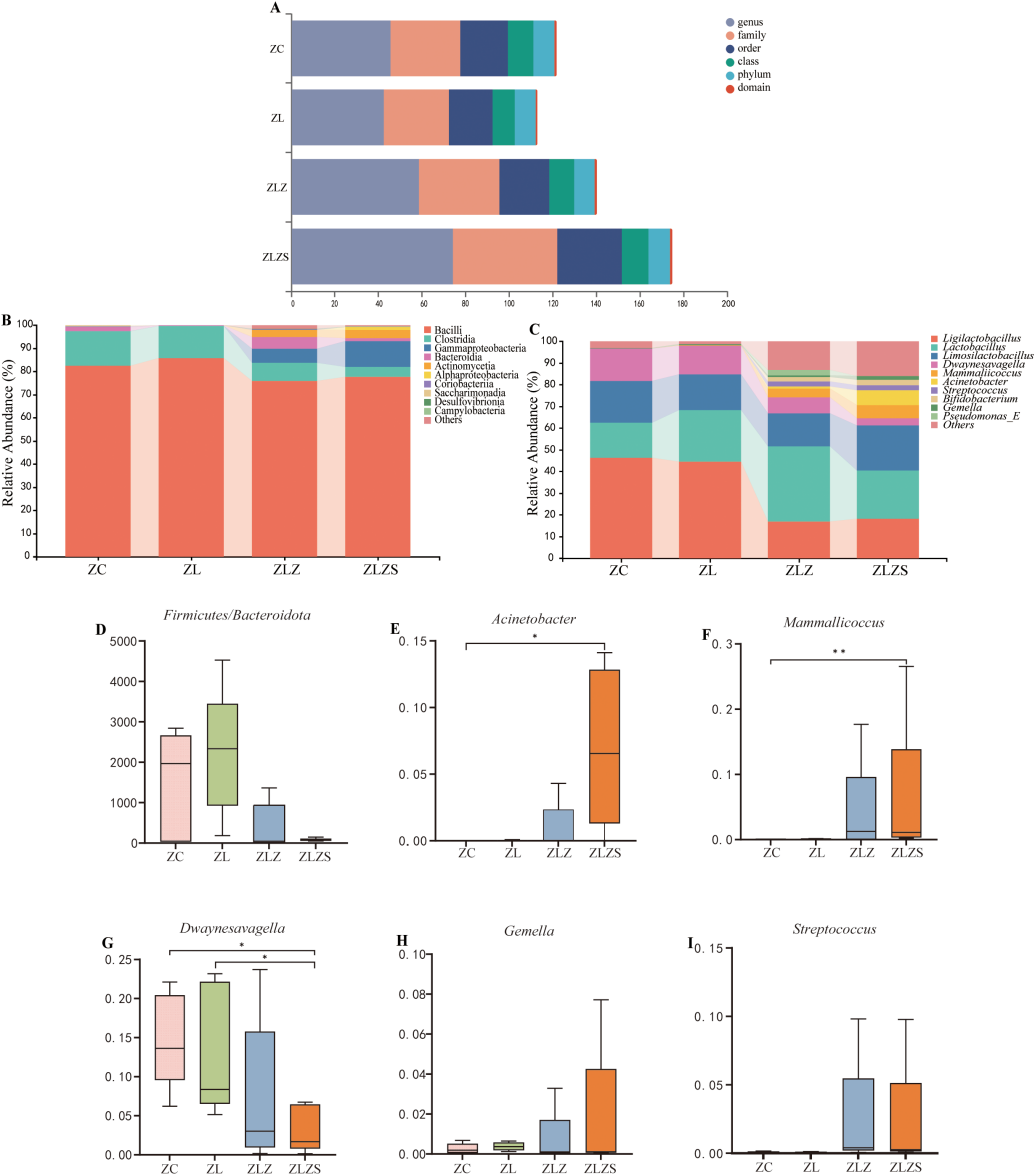
Composition and abundance of the small intestinal microbiota in mice. **(A)** Multiaxis bubble chart showing the overall microbiota composition. **(B)** Phylum-level horizontal bar chart displaying the top 10 dominant phyla ranked by relative abundance. **(C)** Genus-level horizontal bar chart depicting the top 10 dominant genera on the basis of relative abundance. **(D)** Firmicutes/Bacteroidetes (F/B) ratio. Panels **(E–I)** illustrate the dominant genera in the small intestinal contents of each mouse group. Statistical significance is indicated as ^*^ *p*<0.05, ^**^ *p*<0.01. The groups are as follows: ZC (normal group), ZL (standing group), ZLZ (standing combined with lard group), and ZLZS (standing combined with internal and external wet environment group).

Different intervening factors led to variations in the small intestinal microbiota at six taxonomic levels (**Figure 9A**). The top 10 phyla and genera in terms of relative abundance were identified and displayed in histogram form. As illustrated in **Figure 9B**, at the phylum level, *Bacilli*, *Clostridia*, *Gammaproteobacteria*, and *Bacteroidia* were predominant. Compared with that in the ZC group, the abundance of Bacilli decreased in both the ZLZ and ZLZS groups but increased in the ZL group. Additionally, compared with those in the ZLZ group, the abundances of *Clostridia* and *Bacteroidia* decreased, whereas the abundances of *Actinomycetia* and *Gammaproteobacteria* increased in the ZLZS group. **Figure 9D** shows that, relative to that in the ZC group, the Firmicutes/Bacteroidetes (F/B) ratio increased in the ZL group but decreased in the ZLZ and ZLZS groups, although these differences were not statistically significant (*p*>0.05).

**Figure 9C** shows the abundance of the small intestinal microbiota at the genus level. The top 10 most abundant genera were identified, revealing both similarities in composition and differences in abundance across groups. Prominent genera include *Ligilactobacillus*, *Lactobacillus*, *Limosilactobacillus*, *Dwaynesavagella*, *Mammallicoccus*, and *Acinetobacter*. As depicted in **Figures 9E–I**, significant differences were observed in the genus-level bacterial abundances. Specifically, *Acinetobacter* levels were significantly greater in the ZLZS group than in the ZC group (*p*<0.05). Similarly, *Mammallicoccus* abundance was significantly greater in the ZLZS group than in the ZC group (*p*<0.01). Conversely, *Dwaynesavagella* was significantly lower in the ZLZS group than in the ZC and ZL groups (*p*<0.05). No significant differences were found in the abundances of *Gemella* or *Streptococcus* among the groups (*p*>0.05).

### 3.7 Characteristics of the intestinal microbiota in mice with spleen deficiency, dampness syndrome and diarrhea

**Figure 10:**
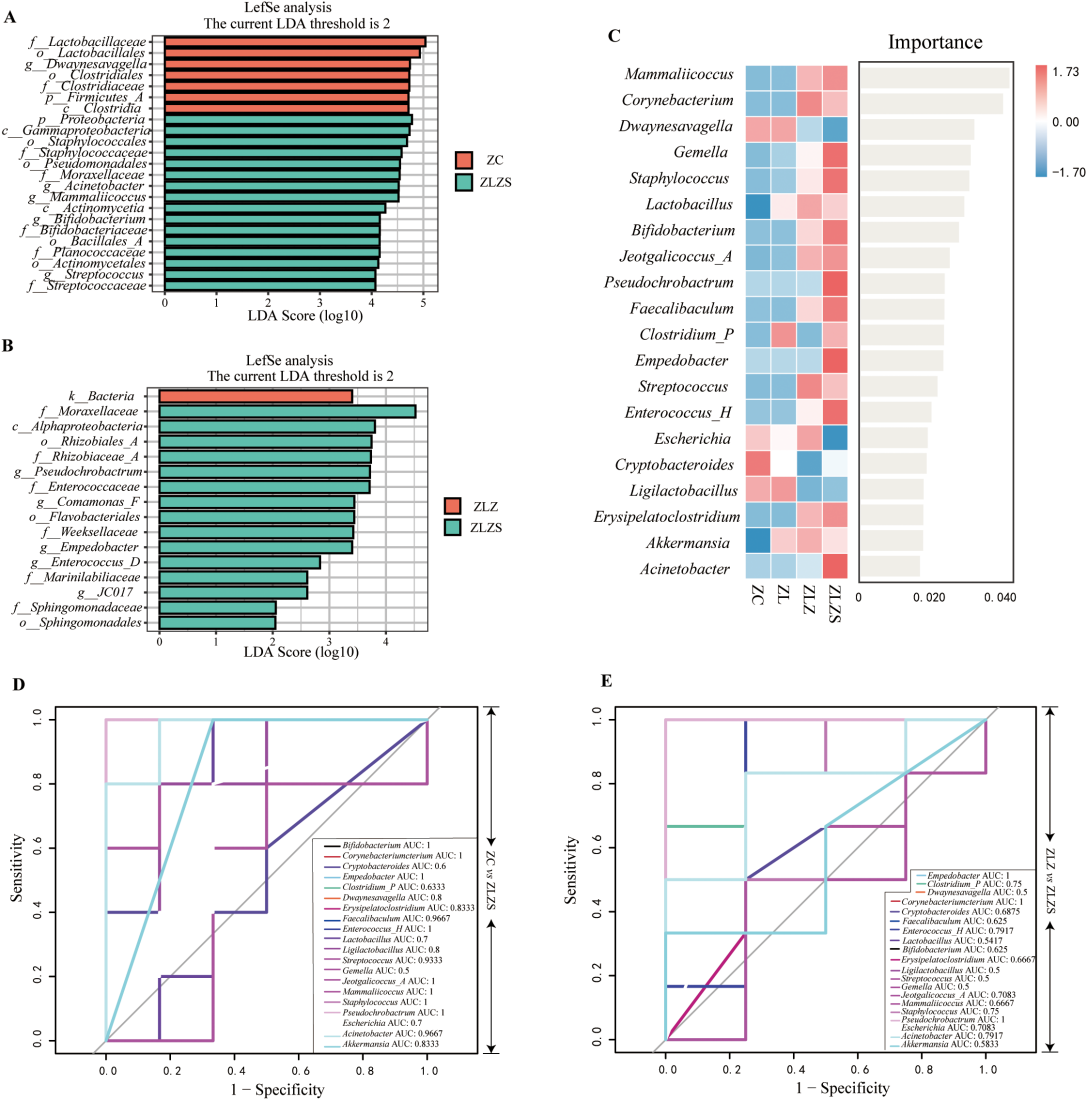
Analysis of the core microbiota in mouse small intestinal contents. **(A)** LDA plot highlighting key genera with significant differences in abundance between ZC group and ZLZS group. **(B)** LDA plot highlighting key genera with significant differences in abundance between ZLZ group and ZLZS group. **(C)** Random forest plot showing the importance of each genus in differentiating the groups. **(D, E)** ROC curves for each genus. **(D)** compares ZC *vs.* ZLZS, and **(E)** compares ZLZ *vs.* ZLZS.

**Figure 10** shows the results of linear discriminant analysis effect size (LEfSe), which was utilized to identify genera with significant differences in abundance among the experimental groups, with a linear discriminant analysis (LDA) score threshold set at >2. This analysis revealed distinct microbial profiles for different groups. **Figure 10A** shows that the ZC group and the ZLZ group, as well as the ZLZ group and the ZLZS group, presented unique microbial community compositions. Specifically, seven genera were identified as key differentiators in the ZC group, whereas sixteen genera were identified in the ZLZS group. Notably, *Dwaynesayagella* was significantly enriched in the ZC group, whereas genera such as *Acinetobacter*, *Mammaliicoccus*, *Bifidobacterium*, and *Streptococcus* were notably enriched in the ZLZS group. **Figure 10B** shows that the ZLZ group had one key differentiating genus, whereas the ZLZS group had fifteen. Genera such as *Pseudochrobactrum*, *Comamonas_F*, *Empedobacter*, *Enterococcus_D*, and *JC017* were significantly enriched in the ZLZS group.

These results underscore the pronounced shifts in microbial community composition associated with different experimental conditions, reflecting the influence of spleen deficiency and dampness syndrome on the intestinal microbiota.

To identify key species distinguishing the ZLZS group, a random forest diagnostic model was constructed using the top 20 enriched genera (**Figure 10C**). This model revealed nonlinear relationships between variables. Genera with an area under the curve (AUC) >0.8 were considered significant (**Figures 10D, E**). Compared with those in the ZC group, the characteristic genera in the ZLZS group included *Bifidobacterium* (AUC=1), *Corynebacterium* (AUC=1), *Empedobacter* (AUC=1), *Dwaynesavagella* (AUC=0.8), *Erysipelatoclostridium* (AUC=0.8333), *Faecalibaculum* (AUC=0.9667), *Enterococcus_H* (AUC=1), *Streptococcus* (AUC=0.9333), *Mammaliicoccus* (AUC=1), *Staphylococcus* (AUC=1), *Acinetobacter* (AUC=0.9667), *Ligilactobacillus* (AUC=0.8), *Jeotgalicoccus_A* (AUC=1), and *Akkermansia* (AUC=0.8333). These genera presented high AUC values, indicating a strong association with the ZLZS group. Compared with the ZLZ group, the ZLZS group had significantly different AUC values for genera such as *Empedobacter* (AUC=1), *Corynebacterium* (AUC=1), and *Pseudochrobactrum* (AUC=1). These findings suggest that, compared with simple lard gavage, the combination of internal and external wet environments enhances the enrichment of these genera. The combination of LEfSe, random forest, and ROC curve analyses identified *Corynebacterium*, *Empedobacter*, and *Pseudochrobactrum* as key members of the characteristic microbiota in the ZLZS model.

### 3.8 The functional impact of a multiplatform water environment combined with a high-fat diet on the intestinal microbiota of mice with diarrhea

**Figure 11:**
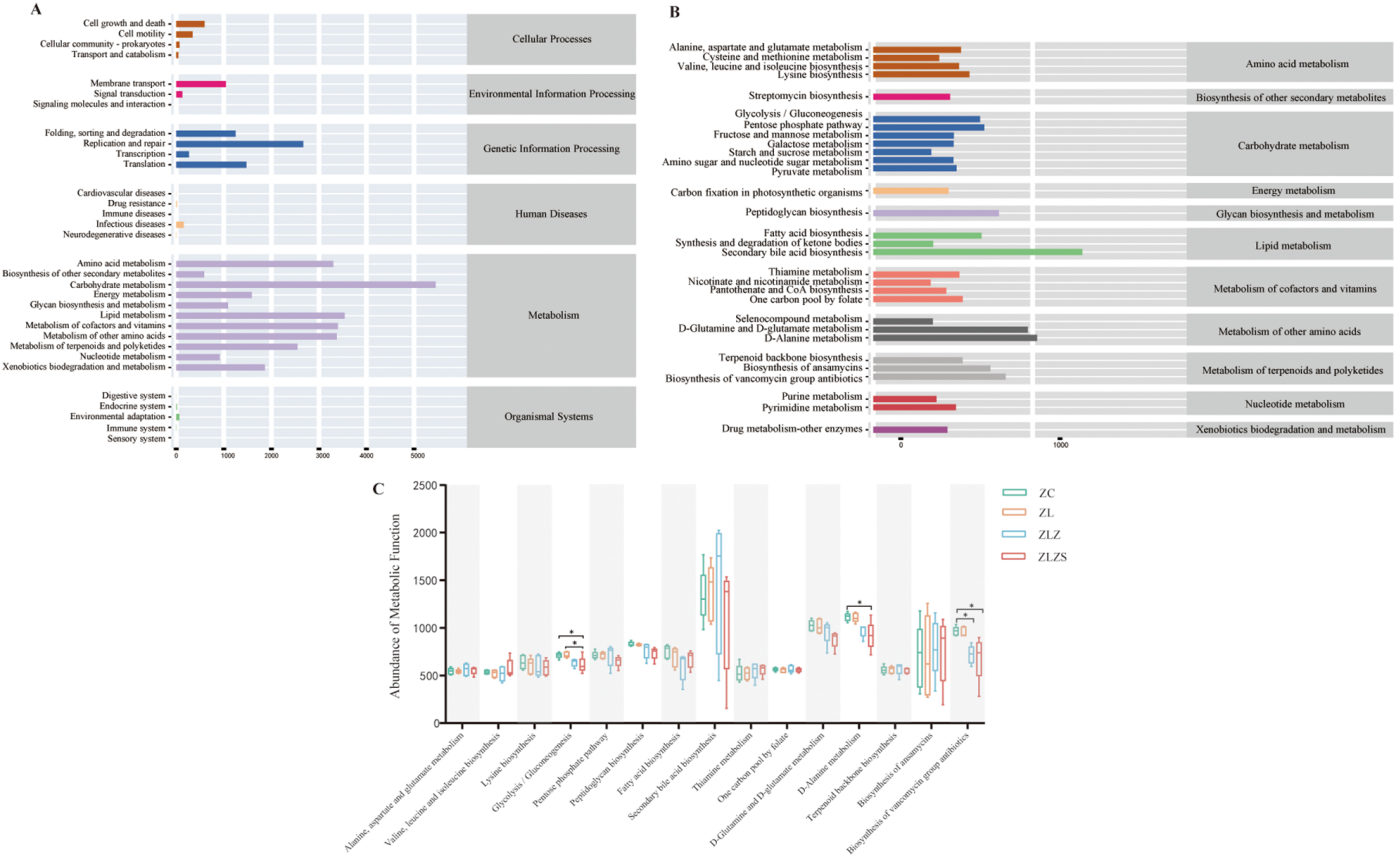
PICRUSt2 prediction and microbial-related metabolic pathways based on the KEGG database. **(A)** KEGG function prediction dependency: the horizontal axis represents the abundance of KEGG functional pathways, whereas the vertical axis denotes the secondary classification of these pathways. The rightmost axis illustrates the primary classification level of the pathways. **(B)** Metabolic pathway abundance: The horizontal axis indicates the abundance of metabolic pathways, the vertical axis represents the third-level classification of these pathways, and the rightmost axis denotes the second-level classification to which each metabolic pathway belongs (median > 356.15975). **(C)** Comparison of metabolic functions across groups: This figure compares the median values of various metabolic pathways between groups, focusing on those with a median greater than 529.969. This highlights significant differences in metabolic functions among the groups. Statistical significance is indicated as follows: *p<0.05

To assess the metabolic and functional changes in the small intestinal microbiota of mice subjected to a high-fat diet combined with internal and external moisture environments, we employed PICRUSt2, which is based on the KEGG database, to predict and analyze microbial metabolic pathways. **Figure 11A** illustrates six primary functional categories (cellular processes, environmental information processing, genetic information processing, human diseases, metabolism, and functional systems) encompassing 32 functional pathways, with metabolism being the most prevalent. Notably, the median metabolic function score for the third metabolic pathway exceeded 356.15975 across the 30 categories. As depicted in **Figure 11B**, the predominant metabolic functions included amino acid metabolism, carbohydrate metabolism, lipid metabolism, energy metabolism, and the metabolism of factors and vitamins.

As shown in **Figure 11C**, we selected third-level metabolic pathways with a median value greater than 529.969 across 15 categories for statistical analysis. Compared with those in the ZC and ZL groups, the activity of the glycolysis/gluconeogenesis pathway in the ZLZS group was significantly lower (*p*<0.05). Additionally, D-alanine metabolism was significantly lower in the ZLZS group than in the ZC group (*p*<0.05). The biosynthesis of vancomycin antibiotics was significantly lower in both the ZLZ and ZLZS groups than in the ZC group (*p*<0.05). Conversely, pathways such as secondary bile acid biosynthesis, thiamine metabolism, and ansamycin biosynthesis tended to increase, although these changes were not statistically significant (*p*>0.05).

In summary, fatigue leads to increased synthesis of secondary bile acids and thiamine while decreasing the biosynthesis of fatty acids and ansamycin. A high-fat diet results in reduced vancomycin antibiotic and lysine biosynthesis in mice but increases the metabolism of alanine, aspartate, and glutamate. An external humid environment decreases the efficiency of glycolysis/gluconeogenesis and D-alanine metabolism, affects energy metabolism, and impacts the biosynthesis of secondary bile acids, ansamycin, and vancomycin antibiotics to some extent. Overall, these factors—fatigue, a high-fat diet, and external moisture—contribute to a general decline in the metabolic function of mice.

### 3.9 Analysis of the correlations between characteristic bacterial genera and metabolism, gastrointestinal function, fluid and energy metabolism

**Figure 12:**
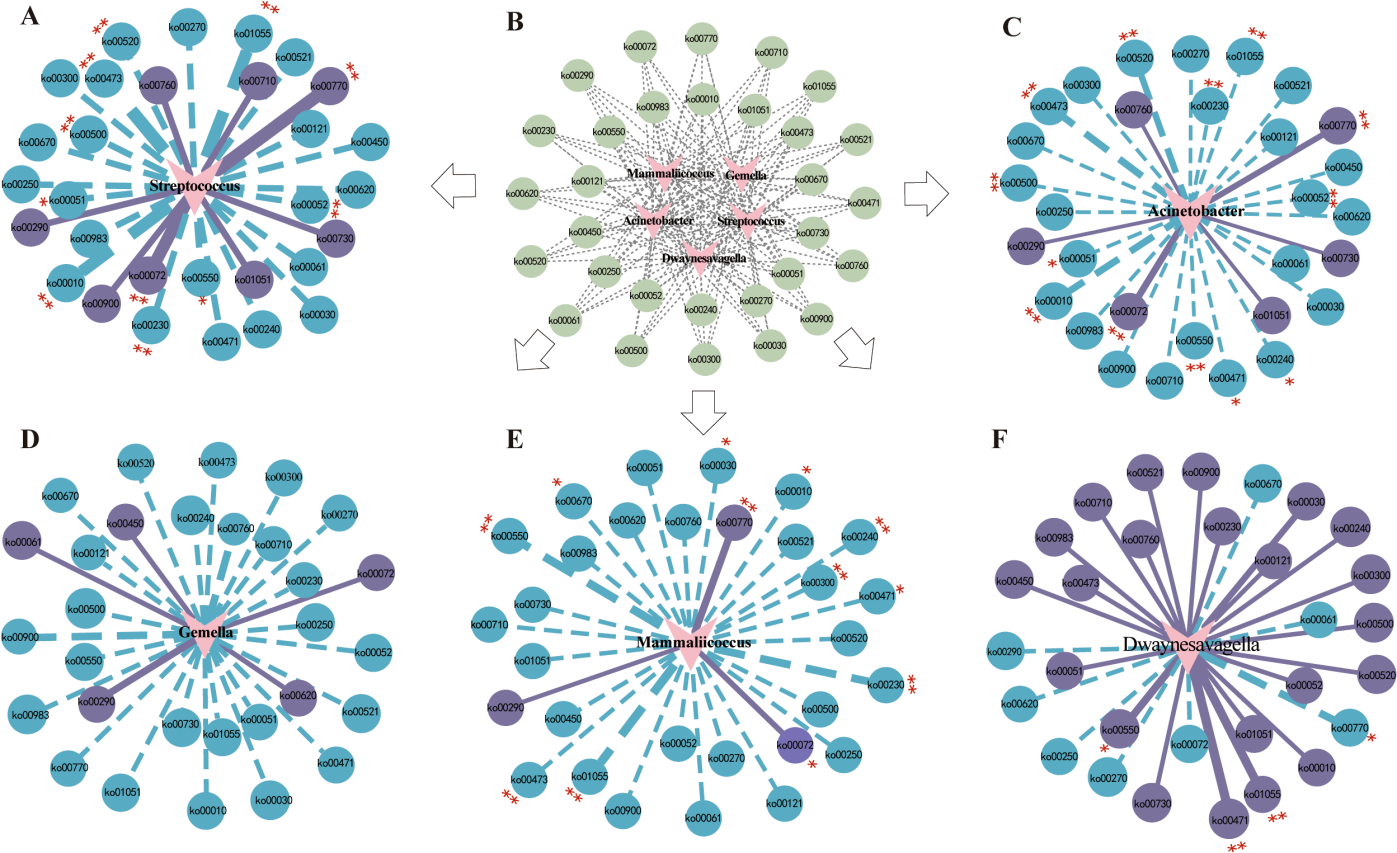
Spearman correlation analysis network diagrams. The solid lines represent positive correlations, whereas the dotted lines denote negative correlations. The thickness of the lines reflects the strength of the correlations. **(A)** Correlation network of *Streptococcus* with metabolic pathways. **(B)** Predictive abundance diagram of functions. **(C)** Correlation network of *Acinetobacter* with metabolic pathways. **(D)** Correlation network of *Gemella* with metabolic pathways. **(E)** Correlation network of *Mammaliicoccus* with metabolic pathways. **(F)** Correlation network of *Dwaynesavagella* with metabolic pathways. Statistical significance is indicated by p values: ^*^ *p*<0.05 and ^**^ *p*<0.01.

To investigate the role of the small intestinal microbiota and metabolic functions in maintaining the stability of the intestinal microenvironment, we performed Spearman correlation analysis between metabolic pathways and key bacterial genera. This analysis revealed significant relationships between specific bacterial genera and metabolic pathways, as illustrated in **Figure 12**.

The correlation network revealed that *Streptococcus* was significantly negatively correlated with D-Alanine metabolism (*p*<0.001, ρ=-0.8466) and significantly positively correlated with Pantothenate and CoA biosynthesis (*p*<0.001, ρ=0.7308). *Acinetobacter* was significantly negatively correlated with D-Alanine metabolism (*p*<0.001, ρ=-0.6906) and significantly positively correlated with Synthesis and degradation of ketone bodies (*p*<0.001, ρ=0.6264). *Mammaliicoccus* was significantly negatively correlated with Biosynthesis of vancomycin group antibiotics (*p*<0.001, ρ=-0.7503) and significantly positively correlated with Pantothenate and CoA biosynthesis (*p*<0.001, ρ=0.6024). *Dwaynesavagella* was significantly positively correlated with D-Glutamine and D-glutamate metabolism (*p*<0.001, ρ=0.8135) and negatively correlated with Pantothenate and CoA biosynthesis (*p*<0.05, ρ=-0.4992). Overall, D-Alanine metabolism, Pantothenate and CoA biosynthesis, Synthesis and degradation of ketone bodies, Biosynthesis of vancomycin group antibiotics, and D-Glutamine and D-glutamate metabolism appear to be the primary pathways influencing the bacterial microbiota in the small intestine of mice.

**Figure 13:**
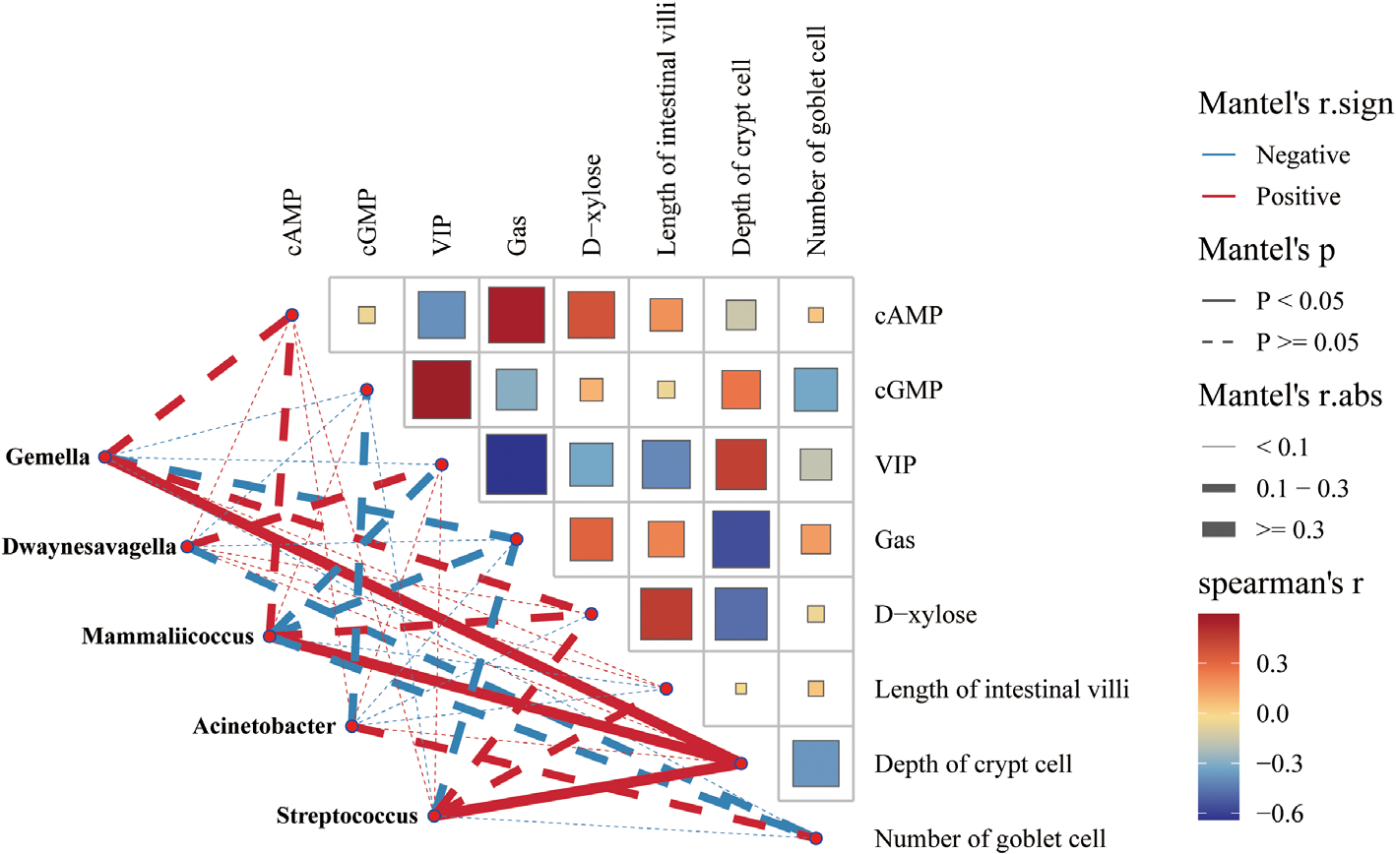
Correlation network heatmap. Nodes: each node represents a variable (*Gemella, Dwaynesavagella, Mammaliicoccus, Acinetobacter, Streptococcus*). Omics heatmap: showing the correlation coefficients between each pair of elements (such as cAMP, cGMP, VIP, and Gas, etc). The label next to each node or element represents the variable’s name. Edges/Connections: the lines connecting nodes indicate correlations. Solid line: indicates a significant correlation. Dashed line: indicates a non-significant correlation. Color gradient: the color of the edges or nodes represents the correlation strength and direction. Blue: negative correlations. Red: positive correlations. Node size: the size of each node reflects its significance or degree of influence within the network. Larger nodes indicate greater significance. Correlation scale: a color gradient scale below the heatmap indicates the range of correlation coefficients from -1 to 1: 1: perfect positive correlation (red); 0: no correlation (white); -1: perfect negative correlation (blue).

To further elucidate the relationships between gastrointestinal function, fluid and energy metabolism, and the intestinal microbiota, we conducted a correlation network heatmap analysis of five genera-level bacterial species alongside various indicators, including Gas, VIP, D-xylose, cAMP, cGMP, intestinal villus length, crypt depth, and the number of goblet cells (**Figure 13**). Our analysis revealed that *Gemella*, *Mammaliicoccus*, and *Streptococcus* were significantly positively correlated with crypt depth. Conversely, *Streptococcus*, *Acinetobacter*, *Gemella*, *Mammaliicoccus*, and *Dwaynesavagella* were negatively correlated with cGMP, VIP, Gas, and the number of goblet cells. Among the environmental factors, Gas and cAMP presented the strongest correlation, followed by crypt depth with VIP and D-xylose with intestinal villus length. Notably, Gas was negatively correlated with the VIP and crypt depth.

These findings suggest that interactions between these factors may constitute a crucial mechanism by which characteristic bacterial genera influence various environmental factors through metabolic pathways, potentially contributing to diarrhea.

## 4 Discussion

Diarrhea is defined by an increased frequency of bowel movements and loose or watery stools. Numerous studies have confirmed that intestinal microecology and energy metabolism play pivotal roles in the onset and progression of diarrhea (Gupta et al., 2020). On one hand, fluid imbalance is a hallmark of diarrhea, resulting from various pathological mechanisms. On the other hand, the pathogenesis of diarrhea, triggered by initiating factors, predominantly involves the regulation of fluid and intestinal microecology (Guan, 2021). Fluid balance is intimately linked to energy cycles. When energy metabolism is impaired, intracellular ion overload leads to homeostatic disruption, and energy synthesis disorders cause cell damage or death, which manifests as diarrhea through increased intestinal fluid secretion (Pongkorpsakol et al., 2017). Excessive energy metabolism may also induce oxidative stress, affecting levels of lactate, urea nitrogen, and key enzyme activities (Zhang et al., 2020). Beneficial intestinal microbiota can metabolize energy byproducts, such as fatty acids, to mitigate oxidative stress and inflammatory responses, thereby enhancing anti-fatigue effects (Guo et al., 2016; Zhang et al., 2016). Moreover, dietary patterns are critical drivers of microbial composition and function (Jha et al., 2018). Research suggests that modifying food intake or altering specific microbial taxa could serve as an effective approach for precision medicine. Changes in intestinal microbiota, to some extent, reflect the biochemical signatures of particular foods (Chen et al., 2020; Wastyk et al., 2021). Thus, addressing diarrhea from the perspective of energy metabolism and diet holds significant therapeutic potential.

An appropriate animal model is essential for advancing the modernization of TCM research. The combination of a multi-platform water environment and lard gavage is a primary method for inducing diarrhea in mice, aimed at interventions that target energy metabolism and dietary factors. Diarrhea induced by this model corresponds to the TCM concept of spleen-deficiency dampness syndrome. The multi-platform water environment setup includes factors such as sleep deprivation, prolonged standing, and high levels of stress, which induce fatigue and mental exhaustion in mice (MaChado et al., 2004; Enoka and Duchateau, 2016). These factors mimic the characteristics of excessive fatigue, spleen deficiency, and weakness associated with spleen-deficiency syndromes. In this model, the ZL group simulates conditions of excessive fatigue, spleen deficiency, and physical exhaustion. Additionally, lard, which represents a high-fat, high-protein diet, induces intestinal microbiota dysbiosis, promotes local inflammation, increases intestinal permeability, and triggers lipopolysaccharide (LPS) production, leading to systemic inflammation (Wang et al., 2020; Ojo et al., 2021). The differences between the ZLZ group (lard gavage) and the ZL group highlight the impact of internal dampness. Our previous research demonstrated that, compared to plant oil, lard gavage exhibits a synergistic effect with *Coriobacteriaceae_UCG-002*, a bacterial family that is significantly negatively correlated with glycolysis/gluconeogenesis pathways, influencing both inflammatory responses and energy metabolism (Qiao et al., 2022). In TCM, dampness is divided into internal and external categories. We found that increasing environmental humidity in the existing model exacerbated diarrhea, further underscoring the role of external dampness in promoting the condition.

In Western medicine, the pathological mechanisms underlying “dampness” are primarily associated with conditions such as obesity, diabetes, and inflammatory immune responses. To better simulate an external damp environment and align with the formation and pathological characteristics of spleen-deficiency dampness syndrome-induced diarrhea in TCM, we introduced a moist bedding environment (50g/100mL) in the ZLZS group. From day seven of modeling, the body weight of mice in the ZLZ group was significantly lower than that of the ZC group (*p*<0.01), whereas the body weight of mice in the ZLZS group gradually exceeded that of the ZC group, suggesting that external humidity could further promote obesity.

Mitochondria, as the foundation of biological function, are critical for various life processes (Song et al., 2023). The obesity, muscle fatigue, and weakness observed in the standing mouse model and following lard gavage are closely linked to mitochondrial dysfunction (Cantó-Santos et al., 2020). A decrease in energy metabolism was reflected in reduced cyclic adenosine monophosphate (cAMP) levels, increased cyclic guanosine monophosphate (cGMP) levels, and a lower cAMP/cGMP ratio—trends consistent with our findings (Li et al., 2022b). The differences observed between the ZLZS group and the ZC group suggest that the external damp environment adversely impacts mitochondrial energy metabolism, further exacerbating spleen deficiency and fatigue. Notably, alterations in cAMP may play a pivotal role in this process. The thymus and spleen, key immune organs, reflect the immune regulatory activity of the mice (Ma et al., 2021). Studies have suggested that an external damp environment can significantly alter the levels of inflammatory factors, leading to immune imbalance. The thymus index of the mice in the ZLZ group was significantly lower than that of the ZC and ZLZS groups (*p*<0.01), while no significant difference in the spleen index was observed between the groups. However, the trend in the indexs for both organs was consistent, indicating that diarrhea associated with spleen deficiency and dampness in TCM may be significantly related to thymic function. Moreover, the external damp environment appears to have somewhat suppressed immune deficiency.

Histological analysis of HE-stained intestinal tissues revealed shortened villi and increased crypt depth in both the ZLZ and ZLZS groups, accompanied by varying degrees of inflammatory cell infiltration. These findings suggest that the combination of a multi-platform water environment and lard gavage induces fatigue in mice and that external dampness may exacerbate energy metabolism dysfunction and immune-related inflammation, thereby aggravating diarrhea-induced damage.

The gastric emptying rate, small intestinal propulsion rate, and ghrelin levels are critical indicators for assessing gastrointestinal motility. Diarrhea is frequently accompanied by a series of morphological changes, including gastrointestinal distension, mucosal congestion and shedding, gastric fundic gland swelling, and hyperplasia of the mucosal muscle (Kelty et al., 2023). Among these changes, serum ghrelin acts on receptors in the smooth muscle of the digestive system, leading to the production of inositol triphosphate, which improves gastric mucosal blood flow and enhances gastrointestinal motility (Ren et al., 2021). The concentration of D-xylose is utilized to evaluate the intestinal absorption capacity of xylose (Ge et al., 2022). In our experiment, mice in the ZLZ and ZLZS groups exhibited significantly greater small intestinal distension than those in the ZC and ZL groups, along with fluid accumulation on the gastric mucosal surface. Further investigations revealed decreased serum ghrelin levels in the ZL, ZLZ, and ZLZS groups, with a pronounced decline observed in the ZLZS group. Concurrently, serum D-xylose levels were significantly lower in the ZLZS group (*p*<0.01). These findings suggest that the multi-platform water environment, combined with lard gavage, induces diarrhea in mice, potentially exerting an antagonistic effect on gastrointestinal function within an external humid environment.

An imbalance in fluid metabolism is another hallmark of diarrhea, serving as a crucial indicator that differentiates it from fatigue and gastrointestinal dysfunction. Vasoactive intestinal peptide (VIP) is an inhibitory neurotransmitter that plays a key role in fluid metabolism and smooth muscle relaxation. Upon binding to its receptors, VIP stimulates the intestinal mucosa, increasing the secretion of water and electrolytes, which reflects microscopic changes in stool consistency (Wu et al., 2014). Studies have demonstrated that VIP antagonists can block corticotropin-releasing factor (CRF)-induced diarrhea in rats (Yakabi et al., 2018). In this experiment, serum VIP levels significantly increased in the ZL, ZLZ, and ZLZS groups, with the ZLZS group exhibiting the most pronounced elevation (*p*<0.01). These findings corroborate results related to fecal water content, intestinal water content, and the density of goblet cells per unit area in the small intestine. This suggests that, in addition to influencing energy metabolism and gastrointestinal function, diarrhea may also be affected by an external humid environment through fluid imbalance.

To further investigate the impact of the multi-platform water environment combined with lard gavage and the external humid environment on the microbial community in the small intestine, inter-group comparisons of relative abundance were conducted. The phyla Firmicutes and Bacteroidetes are dominant groups that help maintain intestinal microbiota balance at the phylum level. The Firmicutes/Bacteroidetes (F/B) ratio is commonly used to assess intestinal homeostasis, with an elevated ratio indicating an unstable or imbalanced microbial community (Sun et al., 2023). Our study revealed that the F/B ratio in the ZL group was higher than in the ZC group, while the ratios in the ZLZ and ZLZS groups were lower than in the ZC group, suggesting that the multi-platform water environment combined with lard gavage disrupts intestinal microecological balance, with the external humid environment playing a facilitative or inhibitory role. Compared to the ZC group, the relative abundances of *Ligilactobacillus* and *Dwaynesavagella* in the ZLZ group exhibited a decreasing trend, whereas the relative abundances of *Mammaliicoccus* and *Streptococcus* increased. Notably, the ZLZS group demonstrated higher relative abundances of *Acinetobacter*, *Gemella*, and *Mammaliicoccus* compared to the ZLZ group. *Ligilactobacillus*, a beneficial bacterium, plays a significant role in host intestinal metabolism and immune activity, and its abundance is closely associated with intestinal health (Chuandong et al., 2024). *Dwaynesavagella* (*Candidatus Savagella gallinarum*), a proposed candidate species of segmented filamentous bacteria (SFB), is a strict anaerobe that adheres tightly to the surface of absorptive intestinal epithelial cells, resisting inflammatory responses. Research has demonstrated that such bacteria can influence intestinal Th17 cells and immunoglobulin A (IgA), penetrating the intestinal mucus layer to closely associate with host cells without invading, thereby playing a role in the treatment of ulcerative colitis (UC) and Crohn’s disease (CD) (Chung et al., 2012; Hedblom et al., 2018; Peng et al., 2023). *Acinetobacter* is a common opportunistic pathogen that colonizes the gastrointestinal tract, skin, respiratory tract, and urogenital system, often causing bacteremia, pneumonia, and urinary infections (Dong et al., 2020). Some studies have suggested that alleviating antibiotic-associated diarrhea is primarily linked to increased relative abundances of *norank_f_Muribaculaceae*, *Erysipelatoclostridium*, and *Clostridium_innocuum_group*, alongside decreased relative abundances of *Staphylococcus* and *Acinetobacter* (Lai et al., 2023). The *Staphylococcaceae* family and the genus *Gemella* encompass several microorganisms of significant clinical or biotechnological importance. The *Staphylococcaceae* family primarily forms two lineages: one includes the genera *Aliicoccus*, *Jeotgalicoccus*, *Nosocomiicoccus*, and *Salinicoccus*, while the other comprises *Macrococcus*, *Mammaliicoccus*, and *Staphylococcus* (Bello et al., 2023). *Staphylococci* can be classified into two main categories based on their ability to produce plasma coagulase: coagulase-negative staphylococci and mammaliicocci, which are part of the Gram-positive bacteria group and carry auxiliary genes characterized by mobile elements that confer selective advantages in antibiotic resistance, colonization, and pathogenicity (Hacker and Carniel, 2001; Michels et al., 2021; Ferhaoui et al., 2023). *Gemella* is a Gram-positive coccus and a facultative anaerobe; in active gastritis and intestinal epithelial metaplasia, *Gemella* and *Streptococcus* exhibit a characteristic negative correlation with glycosylated ceramides linked to cancer and infectious diseases (Peng et al., 2023). In summary, our findings indicate that the multi-platform water environment combined with lard gavage may adversely impact host health by increasing conditional pathogenic bacteria and decreasing beneficial bacteria, while the external humid environment influences microbial abundance and diversity.

Correlation network analysis revealed that five dominant bacterial populations were linked to 30 metabolic pathways, with *Dwaynesavagella* exhibiting a unique relationship with these pathways, indicating that intestinal microbiota may have distinct functional differentiation and synergistic mechanisms in metabolic regulation. The correlation network heatmap illustrated a significant positive correlation between crypt depth and *Gemella*, *Mammaliicoccus*, and *Staphylococcus*, while gas levels exhibited a negative correlation with these genera. Additionally, cAMP and cGMP levels correlated with *Gemella*, *Mammaliicoccus*, and *Acinetobacter*, indicating relationships among these characteristic bacteria, energy metabolism, gastrointestinal function, and fluid metabolism during diarrhea. Research has confirmed that LPS induces bacterial diarrhea in weaned piglets, resulting in a decrease in the abundance of *Gemella* in the gastrointestinal microbiota and antagonistic inflammatory responses that affect intestinal microbiota energy metabolism and stress activity (Li et al., 2024). Based on these results, we infer that the multi-platform water environment combined with lard gavage affects microbial abundance and diversity and may also influence secondary metabolic responses regulated by the microbiota, while the external humid environment exacerbates inter-group differences.

Simultaneously, we observed that the metabolic functions of the microbial community in the small intestine were prominently represented in the functional predictions. Secondary bile acid biosynthesis, associated with lipid metabolism, plays a crucial role in secondary metabolism. Bile acids are essential regulators in various processes, including fat digestion and absorption, energy metabolism, glucose homeostasis, intestinal mucosal integrity, immune response, tumor growth, and bacterial proliferation. In addition to their influence on gastrointestinal motility and intestinal water and mucus secretion, bile acids can also affect the intestinal microbiota both directly and indirectly. The decoupling of the antimicrobial activity of secondary bile acids can alter the characteristic intestinal microbiota (Islam et al., 2011; Wei et al., 2020). Additionally, the regulation of energy homeostasis by bile acids is primarily mediated by the farnesoid X receptor (FXR) and G protein-coupled receptor 5 (TGR5) (Perino and Schoonjans, 2022). Research has shown that secondary bile acids (BAs), such as lithocholic acid (LCA), deoxycholic acid (DCA), glycodeoxycholic acid (GDCA), glycocholic acid (GLCA), and taurolithocholic acid (TLCA) in the feces of patients with ulcerative colitis, exhibit positive correlations with *Butyricicoccus*, *Roseburia*, *Clostridium IV*, *Faecalibacterium*, and *Clostridium XIVb* (Yang et al., 2021). These findings suggest a close relationship among bile acids, the intestinal microbiota, and diarrhea. The secondary bile acid biosynthesis pathway is significant in the modeling approach involving the multi-platform water environment combined with lard gavage, providing both theoretical and experimental support for studying diarrhea from the perspective of the “bile acid-intestinal axis”.

In addition to the “bile acid-intestinal axis” system established in this study, our previous research identified the “renal-intestinal axis” as a biological foundation for stool formation and fluid metabolism, playing a significant role in diarrhea related to spleen deficiency and dampness. Communication between the kidneys and intestines occurs through neurotransmitters, neurotrophic factors, the renin-angiotensin system, and the sympathetic adrenal axis, facilitating feedback on signal fluctuations. This theory strongly supports the close connection between renal and gastrointestinal physiology and pathology, offering insights for the treatment of both renal and gastrointestinal diseases (Zhao et al., 2022). Moreover, the “renal-intestinal axis” is linked to the dynamic feedback of the intestinal microbiota and associated mediating networks. Current research (Zhu et al., 2023) suggests that the mechanism of the “target organ-intestinal-microbiota axis” operates on two fronts: first, the intestinal microbiota secretes small molecules that convey information to target organs or cells via the bloodstream; second, both the intestinal microbiota and its metabolites participate in the physiological activities of target organs through neuro-endocrine-immune network pathways. Some scholars have identified trimethylamine N-oxide (TMAO) (Xie et al., 2024) as a mediating factor, clarifying that the “renal-intestinal axis” mediates inflammatory responses contributing to renal dysfunction and intestinal microecological imbalance. Specific intestinal microbiota and their metabolites can serve as common “signal sources” for both the kidneys and intestines, participating in the regulation of physiological and pathological processes. Research [see (Zhao et al., 2022)] has demonstrated the existence of various symbiotic microbiota in gastrointestinal and renal diseases, including the genera *Bacteroides*, *Proteobacteria*, *Clostridium*, *Escherichia*, *Enterococcus*, and *Klebsiella*. Additionally, immune factors, including interleukin-6 (IL-6), interleukin-1β (IL-1β), tumor necrosis factor-alpha (TNF-α), and transforming growth factor-beta (TGF-β), collectively mediate the inflammatory responses associated with both intestinal and renal diseases.

Moving forward, we will leverage the “bile acid-intestinal axis” in the context of spleen deficiency and dampness-associated diarrhea to investigate the roles of bile acid receptors and secondary bile acids, clarifying the relationship between the “renal-intestinal-microbiota axis” and fluid imbalance post-treatment. This approach will enrich the integrated research framework of “microbiota-syndrome-prescription”.

## 5 Conclusion

In conclusion, the combination of a multi-platform water environment and lard gavage effectively induces diarrhea, with external humidity exacerbating this pathological state. This aggravation is likely related to disruptions in gastrointestinal function, energy metabolism, and fluid homeostasis, particularly involving bacterial taxa such as *Streptococcus*, *Acinetobacter*, *Mammaliicoccus*, *Dwaynesavagella*, and *Gemella*. To better understand these relationships, further longitudinal studies are necessary to clarify the causal connections between these microbial communities and environmental factors.

Additionally, using network pharmacology, we have constructed a visualized analysis of the characteristic microbiota and their associated metabolic pathways, elucidating the microbial mechanisms by which the multi-platform water environment, lard gavage, and external humidity contribute to the worsening of diarrhea. This research also refines the preparation of animal models for spleen deficiency with excessive dampness syndrome, a common concept in TCM. In the future, we aim to enhance the intestinal axis framework in spleen-deficiency and dampness-associated diarrhea and deepen microbiome studies related to syndrome differentiation.

## Data Availability

The datasets presented in this study can be found in online repositories. The names of the repository/repositories and accession number(s) can be found below: https://www.ncbi.nlm.nih.gov/, PRJNA1159995.

## References

[B1] Bai T. T. Yang X. Sun H. W. (2021). Study on the mechanism of TCM mediated aquaporin in treating diarrhea-type irritable bowel syndrome. Mod. J. Integr. Tradit. Chin. West. Med. 30, 2955–2960. doi: 10.3969/j.issn.1008-8849.2021.26.025

[B2] Bello S. Mudassir S. H. Rudra B. Gupta R. S. (2023). Phylogenomic and molecular markers based studies on Staphylococcaceae and Gemella species. Proposals for an emended family Staphylococcaceae and three new families (Abyssicoccaceae fam. nov., Salinicoccaceae fam. nov. and Gemellaceae fam. nov.) harboring four new genera, Lacicoccus gen. nov., Macrococcoides gen. nov., Gemelliphila gen. nov., and Phocicoccus gen. nov. Antonie Van Leeuwenhoek. 116, 937–973. doi: 10.1007/s10482-023-01857-6 37523090

[B3] Borcherding N. Jia W. Giwa R. Field R. L. Moley J. R. Kopecky B. J.. (2022). Dietary lipids inhibit mitochondria transfer to macrophages to divert adipocyte-derived mitochondria into the blood. Cell Metab. 34, 1499–1513.e8. doi: 10.1016/j.cmet.2022.08.010 36070756 PMC9547954

[B4] Cantó-Santos J. Grau-Junyent J. M. Garrabou G. (2020). The impact of mitochondrial deficiencies in neuromuscular diseases. Antioxidants (Basel). 9, 964. doi: 10.3390/antiox9100964 33050147 PMC7600520

[B5] Chen Y. R. Zheng H. M. Zhang G. X. Chen F. L. Chen L. D. Yang Z. C. (2020). High oscillospira abundance indicates constipation and low BMI in the guangdong gut microbiome project. Sci. Rep. 10, 9364. doi: 10.1038/s41598-020-66369-z 32518316 PMC7283226

[B6] Chuandong Z. Hu J. Li J. Wu Y. Wu C. Lai G.. (2024). Distribution and roles of Ligilactobacillus murinus in hosts. Microbiol. Res. 282, 127648. doi: 10.1016/j.micres.2024.127648 38367479

[B7] Chung H. Pamp S. J. Hill J. A. Surana N. K. Edelman S. M. Troy E. B.. (2012). Gut immune maturation depends on colonization with a host-specific microbiota. Cell. 149, 1578–1593. doi: 10.1016/j.cell.2012.04.037 22726443 PMC3442780

[B8] Dong H. Liu B. Li A. Iqbal M. Mehmood K. Jamil T.. (2020). Microbiome analysis reveals the attenuation effect of lactobacillus from yaks on diarrhea via modulation of gut microbiota. Front. Cell Infect. Microbiol. 10. doi: 10.3389/fcimb.2020.610781 PMC7920975 33665171

[B9] Enoka R. M. Duchateau J. (2016). Translating fatigue to Human performance. Med. Sci. Sports Exerc. 48, 2228–2238. doi: 10.1249/MSS.0000000000000929 27015386 PMC5035715

[B10] Ferhaoui N. Tanaka R. Sekizuka T. Kuroda M. Sebaihia M. (2023). Whole genome sequencing and pangenome analysis of Staphylococcus/Mammaliicoccus spp. isolated from diabetic foot ulcers and contralateral healthy skin of Algerian patients. BMC Microbiol. 23, 342. doi: 10.1186/s12866-023-03087-2 37974097 PMC10652506

[B11] Fourie N. H. Wang D. Abey S. K. Creekmore A. L. Hong S. Martin C. G.. (2017). Structural and functional alterations in the colonic microbiome of the rat in a model of stress induced irritable bowel syndrome. Gut Microbes 8, 33–45. doi: 10.1080/19490976.2016.1273999 28059627 PMC5341915

[B12] Gao X. Q. Xun J. T. Zhou S. K. Zhang Y. Zhang L. (2023). Jujubae Fructus alleviates intestinal injury caused by toxic medicinals in Shizao Decoction based on correlation between intestinal flora and host metabolism. China J. Chin. Mater. Med. 48, 2792–2802. doi: 10.19540/j.cnki.cjcmm.20230111.401 37282939

[B13] Ge T. Y. Yao Z. M. Li Y. Chen Y. Zhang M. M. Yang Z. H.. (2022). Therapeutic effect of atractylodes macrocephala koidz grown in qimen on rat model with spleen qi deficiency. J. Yunnan Univ. Chin. Med. 45, 59–64, 69. doi: 10.19288/j.cnki.issn.1000-2723.2022.02.015

[B14] Guan Z. (2021). Metagenomics Study on the Structure and Function of Intestinal Microflora Regulated by Modified Renshen Wumei Decoction in Diarrhea Rats. Chengdu University of Traditional Chinese Medicine, Chengdu.

[B15] Guo X. Meng H. Zhu S. Tang Q. Pan R. Yu S. (2016). Stepwise ethanolic precipitation of sugar beet pectins from the acidic extract. Carbohydr Polym. 136, 316–321. doi: 10.1016/j.carbpol.2015.09.003 26572361

[B16] Gupta A. Saha S. Khanna S. (2020). Therapies to modulate gut microbiota: past, present and future. World J. Gastroenterol. 26, 777–788. doi: 10.3748/wjg.v26.i8.777 32148376 PMC7052537

[B17] Hacker J. Carniel E. (2001). Ecological fitness, genomic islands and bacterial pathogenicity. A Darwinian view of the evolution of microbes. EMBO Rep. 2, 376–381. doi: 10.1093/embo-reports/kve097 11375927 PMC1083891

[B18] Hedblom G. A. Reiland H. A. Sylte M. J. Johnson T. J. Baumler D. J. (2018). Segmented filamentous bacteria - metabolism meets immunity. Front. Microbiol. 9. doi: 10.3389/fmicb.2018.01991 PMC6117376 30197636

[B19] Islam K. B. Fukiya S. Hagio M. Fujii N. Ishizuka S. Ooka T.. (2011). Bile acid is a host factor that regulates the composition of the cecal microbiota in rats. Gastroenterology. 141, 1773–1781. doi: 10.1053/j.gastro.2011.07.046 21839040

[B20] Jha A. R. Davenport E. R. Gautam Y. Bhandari D. Tandukar S. Ng K. M.. (2018). Gut microbiome transition across a lifestyle gradient in himalaya. PloS Biol. 16, e2005396. doi: 10.1371/journal.pbio.2005396 30439937 PMC6237292

[B21] Kelty T. J. Taylor C. L. Wieschhaus N. E. Thorne P. K. Amin A. R. Mueller C. M.. (2023). Western diet-induced obesity results in brain mitochondrial dysfunction in female Ossabaw swine. Front. Mol. Neurosci. 16. doi: 10.3389/fnmol.2023.1320879 PMC10755880 38163062

[B22] Lai Y. Deng H. Fang Q. Ma L. Lei H. Guo X.. (2023). Water-insoluble polysaccharide extracted from poria cocos alleviates antibiotic-associated diarrhea based on regulating the gut microbiota in mice. Foods. 12, 3080. doi: 10.3390/foods12163080 37628079 PMC10453245

[B23] Li Z. Y. Li L. Y. Zhang Z. Y. Qiu Y. Zhao F. Dai M.. (2024). Effect of gegen qinlian decoction combined with probiotics on intestinal microbiota and related inflammatory factors in mice with ulcerative colitis. Cent. South Pharm. 22, 92–100. doi: 10.7539/j.issn.1672-2981.2024.01.014

[B24] Li X. Y. Peng X. X. Qiao B. Peng M. J. Deng N. Yu R.. (2022a). Gut-kidney impairment process of adenine combined with folium sennae-induced diarrhea: association with interactions between lactobacillus intestinalis, bacteroides acidifaciens and acetic acid, inflammation, and kidney function. Cells. 11, 3261. doi: 10.3390/cells11203261 36291135 PMC9599973

[B25] Li C. Wang Y. Zhao X. Li J. Wang H. Ren Y.. (2024). Comparative analysis of intestinal inflammation and microbiota dysbiosis of LPS-challenged piglets between different breeds. Anim. (Basel). 14, 665. doi: 10.3390/ani14050665 PMC10930963 38473050

[B26] Li X. Y. Zhu J. Y. Wu Y. Liu Y. W. Hui H. Y. Tan Z. J. (2022b). Model building and validation of diarrhea mice with kidney-yang depletion syndrome. J. Tradit. Chin. Med. 63, 1368–1373. doi: 10.13288/j.11-2166/r.2022.14.012

[B27] Liu J. Qiao B. Deng N. Wu Y. Li D. Tan Z. J. (2023). The diarrheal mechanism of mice with a high-fat diet in a fatigued state is associated with intestinal mucosa microbiota. 3 Biotech. 13, 77. doi: 10.1007/s13205-023-03491-5 PMC9902584 36761339

[B28] Ma T. Li C. Zhao F. Cao J. Zhang X. Shen X. (2021). Effects of co-fermented collagen peptide-jackfruit juice on the immune response and gut microbiota in immunosuppressed mice. Food Chem. 365, 130487. doi: 10.1016/j.foodchem.2021.130487 34237564

[B29] Ma P. J. Wang M. M. Wang Y. (2022). Gut microbiota: A new insight into lung diseases. Biomed. Pharmacother. 155, 113810. doi: 10.1016/j.biopha.2022.113810 36271581

[B30] MaChado R. B. Hipólide D. C. Benedito-Silva A. A. Tufik S. (2004). Sleep deprivation induced by the modified multiple platform technique: quantification of sleep loss and recovery. Brain Res. 1004, 45–51. doi: 10.1016/j.brainres.2004.01.019 15033418

[B31] Michels R. Last K. Becker S. L. Papan C. (2021). Update on coagulase-negative staphylococci-what the clinician should know. Microorganisms. 9, 830. doi: 10.3390/microorganisms9040830 33919781 PMC8070739

[B32] Mu J. X. (2023). Clinical Observation of Shenzhu Zhixie Decoction in the Treatment of Antibiotic Associated Diarrhea in Children (Spleen and Stomach Deficiency Syndrome). Hebei University of Traditional Chinese Medicine, Shijiazhuang(Hebei.

[B33] Ojo O. Ojo O. O. Zand N. Wang X. (2021). The effect of dietary fibre on gut microbiota, lipid profile, and inflammatory markers in patients with type 2 diabetes: A systematic review and meta-analysis of randomised controlled trials. Nutrients. 13, 1805. doi: 10.3390/nu13061805 34073366 PMC8228854

[B34] Peng L. Guo Y. Gerhard M. Gao J. J. Liu Z. C. Mejías-Luque R.. (2023). Metabolite alterations and interactions with microbiota in *helicobacter pylori*-associated gastric lesions. Microbiol. Spectr. 11, e0534722. doi: 10.1128/spectrum.05347-22 37358459 PMC10434277

[B35] Peng X. X. Yi X. Deng N. Liu J. Tan Z. J. Cai Y. (2023). Zhishi Daozhi decoction alleviates constipation induced by a high-fat and high-protein diet via regulating intestinal mucosal microbiota and oxidative stress. Front. Microbiol. 14. doi: 10.3389/fmicb.2023.1214577 PMC10544343 37789856

[B36] Perino A. Schoonjans K. (2022). Metabolic Messengers: bile acids. Nat. Metab. 4, 416–423. doi: 10.1038/s42255-022-00559-z 35338368

[B37] Pongkorpsakol P. Yimnual C. Chatsudthipong V. Rukachaisirikul V. Muanprasat C. (2017). Cellular mechanisms underlying the inhibitory effect of flufenamic acid on chloride secretion in human intestinal epithelial cells. J. Pharmacol. Sci. 134, 93–100. doi: 10.1016/j.jphs.2017.05.009 28651800

[B38] Qiao B. Li X. Y. Wu Y. Guo T. Tan Z. J. (2022). Comparative Analysis of the Gut microbiota in Mice under Lard or Vegetable blend Oil Diet. J. Oleo Sci. 71, 1613–1624. doi: 10.5650/jos.ess22056 36198580

[B39] Qiao B. Liu J. Li D. D. Li X. Y. Liu Y. W. Tan Z. J. (2023). Comparative study on five modeling methods of spleen qi deficiency syndrome based on the theory that “Diet and fatigue damage the spleen. J. Tradit. Chin. Med. 64, 1149–1156. doi: 10.13288/j.11-2166/r.2023.11.013

[B40] Qiao B. Xiao N. Q. Deng N. Tan Z. J. (2024). Shenling Baizhu powder attenuates lard diet in a fatigued state-induced diarrhea via targeting microbial metabolites short chain fatty acids-mediated lipid metabolism. 3 Biotech. 14, 203. doi: 10.1007/s13205-024-04045-z PMC11329475 39157421

[B41] Qiu P. X. Feng W. P. Zhou Y. Zhang J. F. (2020). Effects of exercise on the characteristics of human energy metabolism under hot and different humidity environment. Chin. J. Sports Med. 39, 932–936. doi: 10.16038/j.1000-6710.2020.12.003

[B42] Ren X. Wang Y. He Z. Liu H. Xue K. (2021). Effects of cefuroxime axetil combined with Xingpi Yanger granules on the serum gastrin, motilin, and somatostatin levels in children with upper respiratory tract infection accompanied by diarrhea: results of a randomized trial. Transl. Pediatr. 10, 2106–2113. doi: 10.21037/tp-21-314 34584881 PMC8429862

[B43] Shao H. Q. He Y. S. Xiao N. Q. Xie G. Z. Tan Z. J. (2022). Establishment of a mouse model of diarrhea with gastrointestinal food stagnation syndrome and the efficacy of baohe wan. Lishizhen Med. Mater. Med. 33, 10–15. doi: 10.3969/j.issn.1008-0805.2022.01.03

[B44] Sharma B. Sengupta T. Chandra Vishwakarma L. Akhtar N. Mallick H. N. (2021). Muscle temperature is least altered during total sleep deprivation in rats. J. Therm Biol. 98, 102910. doi: 10.1016/j.jtherbio.2021.102910 34016337

[B45] Shen Y. Y. (2021). Study on the Effect of Acupuncture on Atherosclerosis and Intestinal Microflora in Mice Based on"Heart and Small Intestine. Guangxi University of Traditional Chinese Medicine, Nanning(Guangxi.

[B46] Song Y. X. Wang J.l. Zheng Z.l. Wei W. L. Zhang W. P. Feng T.. (2023). The effect of external humidity on mitochondrial autophagy in normal and rheumatoid. Prog. Mod. Biomed. 23, 417–422, 493. doi: 10.13241/j.cnki.pmb.2023.03.004

[B47] Sun T. Liu X. Su Y. Wang Z. Cheng B. Dong N.. (2023). The efficacy of anti-proteolytic peptide R7I in intestinal inflammation, function, microbiota, and metabolites by multiomics analysis in murine bacterial enteritis. Bioeng Transl. Med. 8, e10446. doi: 10.1002/btm2.10446 36925697 PMC10013768

[B48] Tang Y. S. Kong J. Zhou B. D. Xiong G. S. Li F. L. Wang X. S. (2021). The role of intestinal microbiota in the progression of severe acute pancreatitis and its potential therapeutic implications. Chin. J. Microecol. 33, 980–984. doi: 10.13381/j.cnki.cjm.202108024

[B49] Tong Y. Gao H. Qi Q. Liu X. Li J. Gao J.. (2021). High fat diet, gut microbiome and gastrointestinal cancer. Theranostics. 11, 5889–5910. doi: 10.7150/thno.56157 33897888 PMC8058730

[B50] Wang R. C. (2022). Pathological and Physiological Basis of Central Fatigue Syndrome of Spleen Deficiency and Dampness Excess and Mechanism of Shenlingbaizhu Powder's Intervention. Beijing University of Traditional Chinese Medicine, Beijing.

[B51] Wang B. Kong Q. Li X. Zhao J. Zhang H. Chen W.. (2020). A high-fat diet increases gut microbiota biodiversity and energy expenditure due to nutrient difference. Nutrients. 12, 3197. doi: 10.3390/nu12103197 33092019 PMC7589760

[B52] Wang R. C. Wu F. Z. Dai N. Jiang Y. Liao W. Y. Zhang W. Y.. (2024). Construction method of animal model of spleen deficiency and dampness excess syndrome. World Chin. Med. 19, 88–93, 99. doi: 10.3969/j.issn.1673-7202.2024.01.017

[B53] Wastyk H. C. Fragiadakis G. K. Perelman D. Dahan D. Merrill B. D. Yu F. B.. (2021). Gut-microbiota-targeted diets modulate human immune status. Cell. 184, 4137–4153.e14. doi: 10.1016/j.cell.2021.06.019 34256014 PMC9020749

[B54] Wei W. Wang H. F. Zhang Y. Zhang Y. L. Niu B. Y. Yao S. K. (2020). Altered metabolism of bile acids correlates with clinical parameters and the gut microbiota in patients with diarrhea-predominant irritable bowel syndrome. World J. Gastroenterol. 26, 7153–7172. doi: 10.3748/wjg.v26.i45.7153 33362374 PMC7723672

[B55] Wu Y. Peng X. X. Li X. Y. Li D. D. Tan Z. J. Yu R. (2022). Sex hormones influence the intestinal microbiota composition in mice. Front. Microbiol. 13. doi: 10.3389/fmicb.2022.964847 PMC9659915 36386696

[B56] Wu R. M. Sun Y. Y. Zhou T. T. Zhu Z. Y. Zhuang J. J. Tang X.. (2014). Arctigenin enhances swimming endurance of sedentary rats partially by regulation of antioxidant pathways. Acta Pharmacol. Sin. 35, 1274–1284. doi: 10.1038/aps.2014.70 25152028 PMC4186987

[B57] Wu Y. Zhang C. Y. Shao H. Q. Luo H. H. Tan Z. J. (2021). Characteristics of intestinal microbiota and enzyme activities in mice fed with lily bulb. 3 Biotech. 11, 17. doi: 10.1007/s13205-020-02597-4 PMC7778670 33442516

[B58] Xie S. Q. Deng N. Fang L. Y. Shen J. X. Tan Z. J. Cai Y. (2024). TMAO is involved in kidney-yang deficiency syndrome diarrhea by mediating the "gut-kidney axis. Heliyon. 10, e35461. doi: 10.1016/j.heliyon.2024.e35461 39170478 PMC11336722

[B59] Xie G. Z. Tang Y. Huang L. L. Tan Z. J. (2021). Effects of total glycosides of qiwei baizhu powder on intestinal microbiota and enzyme activities in diarrhea mice. Biotechnol. Bull. 37, 124–131. doi: 10.13560/j.cnki.biotech.bull.1985.2021-0149

[B60] Yakabi S. Wang L. Karasawa H. Yuan P. Q. Koike K. Yakabi K.. (2018). VIP is involved in peripheral CRF-induced stimulation of propulsive colonic motor function and diarrhea in male rats. Am. J. Physiol. Gastrointest Liver Physiol. 314, G610–G622. doi: 10.1152/ajpgi.00308.2017 29420068 PMC6008061

[B61] Yan Y. P. (2023). Study on the Effect and Mechanism of MAPK4 Knockout on Inflammatory Bowel Disease in Mice. Guizhou Medical University, Zunyi(Guizhou.

[B62] Yang Z. H. Liu F. Zhu X. R. Suo F. Y. Jia Z. J. Yao S. K. (2021). Altered profiles of fecal bile acids correlate with gut microbiota and inflammatory responses in patients with ulcerative colitis. World J. Gastroenterol. 27, 3609–3629. doi: 10.3748/wjg.v27.i24.3609 34239273 PMC8240054

[B63] Yang M. Ngo W. Srinivasan S. Heynen M. L. Dantam J. Subbaraman L. N.. (2021). Optimization of goblet cell density quantification methods. Exp. Eye Res. 207, 108607. doi: 10.1016/j.exer.2021.108607 33930401

[B64] Zhang Z. Guo L. Yan A. Feng L. Wan Y. (2020). Fractionation, structure and conformation characterization of polysaccharides from anoectochilus roxburghii. Carbohydr Polym. 231, 115688. doi: 10.1016/j.carbpol.2019.115688 31888812

[B65] Zhang Q. Wu Y. Wang J. Wu G. Long W. Xue Z.. (2016). Accelerated dysbiosis of gut microbiota during aggravation of DSS-induced colitis by a butyrate-producing bacterium. Sci. Rep. 6, 27572. doi: 10.1038/srep27572 27264309 PMC4893749

[B66] Zhao Y. Niu R. N. Ma Y. G. Zhang X. Zhao S. L. (2022). Research progress of brain-kidney-gut axis. Acta Chin. Med. Pharmacol. 50, 88–95. doi: 10.19664/j.cnki.1002-2392.220189

[B67] Zhou M. S. Li X. Y. Liu J. Wu Y. Tan Z. J. Deng N. (2024). Adenine's impact on mice's gut and kidney varies with the dosage administered and relates to intestinal microorganisms and enzyme activities. 3 Biotech. 14, 88. doi: 10.1007/s13205-024-03959-y PMC10884393 38406640

[B68] Zhu J. P. Peng T. Liu Y. Zi L. L. Ma X. H. Tian Y.. (2023). The mechanism of action of zhang zhen's "One body, two wings, and qi regulation" Therapy for irritable bowel syndrome based on gut microbiota. Lishizhen Med. Mater. Med. 34, 1984–1987. doi: 10.3969/j.issn.1008-0805.2023.08.51

[B69] Zhu M. M. Wang L. X. Feng R. Y. Zhang Z. J. Zhao T. C. (2021). Ge,S.Q. Construction and evaluation of a mouse model of simple obesity with spleen deficiency and damp stagnation. J. Basic Chin. Med. 27, 247–250. doi: 10.19945/j.cnki.issn.1006-3250.2021.02.018

